# Optimizing the fabrication of a 3D high-resolution implant for neural stimulation

**DOI:** 10.1186/s13036-023-00370-8

**Published:** 2023-08-24

**Authors:** Gal Shpun, Nairouz Farah, Yoav Chemla, Amos Markus, Tamar Azrad Leibovitch, Erel Lasnoy, Doron Gerber, Zeev Zalevsky, Yossi Mandel

**Affiliations:** 1 The Alexander Kofkin Faculty of Engineering, Bar Ilan University, 5290002 Ramat Gan, Israel; 2 Faculty of Life Sciences, School of Optometry & Visual Science, Bar Ilan University, 5290002 Ramat Gan, Israel; 3 Bar Ilan Institute for Nanotechnology & Advanced Materials (BINA), Bar Ilan University, 5290002 Ramat Gan, Israel; 4 The Gonda Multidisciplinary Brain Research Center, Bar-Ilan University, Ramat Gan, Israel

**Keywords:** Neural interfaces, Retinal prostheses, Implantable devices, Electrical Neuro-stimulation, SU-8 Photolithography, Bio-MEMS

## Abstract

**Background:**

Tissue-integrated micro-electronic devices for neural stimulation hold great potential in restoring the functionality of degenerated organs, specifically, retinal prostheses, which are aimed at vision restoration. The fabrication process of 3D polymer-metal devices with high resolution and a high aspect-ratio (AR) is very complex and faces many challenges that impair its functionality.

**Approach:**

Here we describe the optimization of the fabrication process of a bio-functionalized 3D high-resolution 1mm circular subretinal implant composed of SU-8 polymer integrated with dense gold microelectrodes (23μm pitch) passivated with 3D micro-well-like structures (20μm diameter, 3μm resolution). The main challenges were overcome by step-by-step planning and optimization while utilizing a two-step bi-layer lift-off process; bio-functionalization was carried out by N_2_ plasma treatment and the addition of a bio-adhesion molecule.

**Main results:**

*In-vitro* and *in-vivo* investigations, including SEM and FIB cross section examinations, revealed a good structural design, as well as a good long-term integration of the device in the rat sub-retinal space and cell migration into the wells. Moreover, the feasibility of subretinal neural stimulation using the fabricated device was demonstrated *in-vitro* by electrical activation of rat’s retina.

**Conclusions:**

The reported process and optimization steps described here in detail can aid in designing and fabricating retinal prosthetic devices or similar neural implants.

**Supplementary Information:**

The online version contains supplementary material available at 10.1186/s13036-023-00370-8.

## Introduction

Prosthetic stimulation to restore various organ functions is currently translated from the bench to the clinic in many fields such as retinal prostheses [1, 2] which are already being clinically evaluated; engineered cardiac tissue, which is in advanced research and developmental stages [3], and deep brain stimulation (DBS) for treating various neuronal diseases, which is already in clinical use [4]. These advanced devices usually require the implantation of flexible electronic implants that enable their integration with the nervous system and are widely investigated [5–7]. However, the fabrication process of such embedded devices is very challenging, since it is based on complex sequential photolithography steps [8], followed by thin-layer metal deposition, lift-off processes, etching [9] and the need for an additional passivation layer that serves as an electrode encapsulation [10].

This work is aimed at optimizing the fabrication process of a high-density implantable device. As a conceptual device, we fabricated a sub-retinal implant composed of gold electrodes deposited on epoxy SU-8, which is widely used as a substrate material in diverse bio-micro-electromechanical system (bio-MEMS) fields due to its mechanical properties and high aspect ratio capabilities. The fabrication process of a free-standing [11, 12] complex 3D [13–15] metal coated [16, 17] bio-electronic device [6, 15, 18–21] poses many challenges. Among the specific main challenges are the creation of 3D structures with a high aspect ratio [14, 22] at a high-resolution, the shaping of metal microelectrode edges while avoiding "ear patterning”, the fabrication of high-density metal microelectrodes with strong adhesion to SU-8, proper release of the device, and the bio-functionalization of the electrodes aimed at enhancing cell adhesion, and encapsulation.

Here, we report and summarize the detailed optimization and troubleshooting of the various steps needed for fabricating a 3D high-resolution implant while addressing various challenges. Finally, as a proof of concept, we present the results of both *ex-vivo* and *in-vivo* experiments by studying the implant integration with the host rat retina and its ability to electrically stimulate the retinal neurons. This work can assist in optimizing the fabrication process of high-density, high aspect ratio, and implementable bioelectronics devices, and contribute to the field of bio-MEMS in general and neurostimulation in particular.

## Materials and methods

### General considerations

**Fig. 1:**
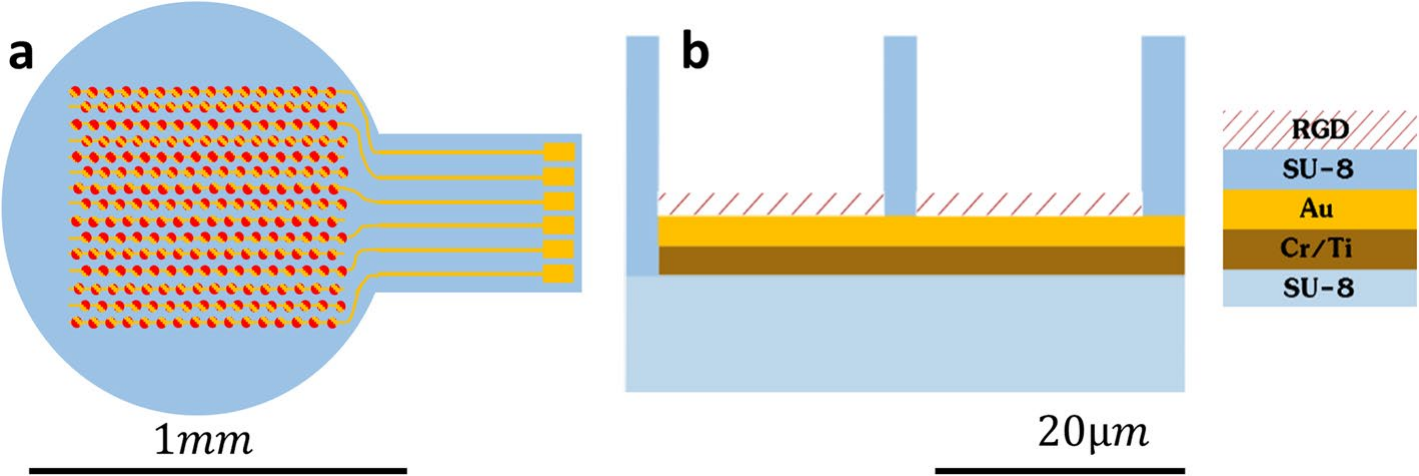
Schematic illustration of the subretinal implant. The implant is composed of an SU-8 substrate and is designed as a 1 mm circular device constructed of 1,020 micro-wells (20 µm in both diameter and height) with a gold electrode at the bottom for neural activation. **a**) Top view of a complete implant structure. **b**) Side view, SU-8 micro-well-like structures; height 17 µm with the gold electrode at the bottom

Our conceptual device (Fig. 1) is a 3D polymeric 1mm circular-shaped implant composed of 1,020 micro-wells (20µm in diameter and height); at the bottom of each well is a gold electrode centrally located for neuronal electrical stimulation. The fabrication of such devices entails a complex layer-by-layer process faced with many challenges; the most critical are thermal stress formation, the need for strong and stable bonding between the inherent gold and the SU-8 epoxy polymer, obtaining a high resolution feature size in the SU-8 (down to 3µm), a high aspect ratio (3:20) geometry built on a surface with varying refractive indices, the proper release of the complete implant from the wafer, and the bio-functionalization of the implant and the gold electrodes to improve neuronal coupling. The design considerations in terms of choice of material and the polymerization parameters for each step will be described in the Results section. In this conceptual device the odd and even rows are alternately connected, allowing for future research of stimulation resolution. A localized retinal stimulation by an *ex-vivo* high-resolution prototype is also described.

### Material selection

To serve as the implant’s main substrate, the epoxy negative photoresist polymer SU-8 (MicroChem, Westborough, MA, USA) [23] was chosen due to its suitable mechanical properties (4-5Gpa) [24, 25], wide range of aspect ratio (AR), biocompatibility, and its increasingly widespread use as a bioMEMS [21, 24] substrate and neuronal growth scaffold [6, 26]. As for the electrode material, gold was chosen due to its chemically inherent, low electrical resistivity, biocompatibility [18, 19] and wide use in bio-MEMS in general, and in retinal prostheses in particular [20, 27, 28].

All photolithography steps were performed by a mask aligner (Karl SUSS MA 6, Germany) using a quartz photomask and the vacuum contact mode. The photoresist development was obtained by sequential immersion in SU-8 developer/IPA and in AZ351/DDW for SU-8 and AZ1505, respectively.

### The main steps in the fabrication process - General description

**Fig. 2:**
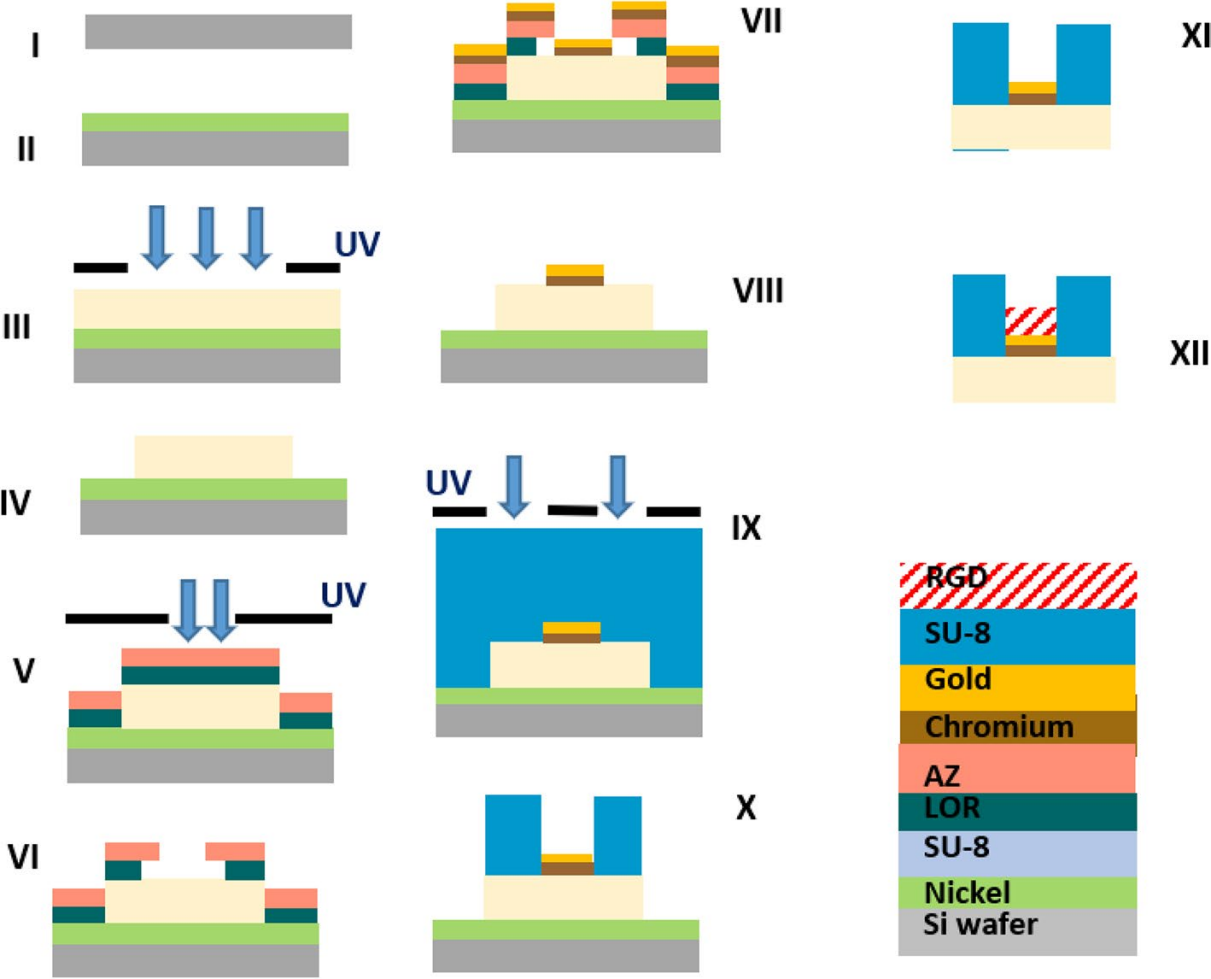
Illustration of the final optimized fabrication process of a SU-8-gold high resolution, high aspect-ratio device. **I**) Ni thin-layer deposition. **II**) SU-8 spin coating, soft bake. **III**) UV exposure and PEB. **IV**) Development (PGMEA), curing and O_2_ plasma. **V**) LOR spin coating, baking, AZ photoresist spin coating, soft bake, and UV exposure. **VI**) AZ development (AZ351, AZ curing, and LOR development. **VII**) O_2_ plasma, Ar ion-milling, and Cr/Au (10/200 nm) metallization by thin-layer sputter deposition. **VIII**) Bi-layer lift-off (NMP). **IX**) 2^nd^ SU8 layer spin coating, soft bake, and UV exposure. **X**) PEB, SU-8 development (PGMEA) and curing, **XI**) wet etch release (HNO_3_), and **XII**) RGD bio-functionalization by immersion

The device fabrication is based on a sequential process of conventional photolithography, which is widely used in the semiconductor and MEMS industry and enables shape writing by photo-sensitive materials (photoresist), through exposure to UV patterns using a photomask [9, 29]. A detailed description of the final optimized process is depicted in Fig. 2.

The first step in the fabrication process is the formation of the implant base by spin coating SU-8 on an oxide silicon wafer (Silicon Specialists, Hayward, CA, USA), which is then illuminated by patterned UV light (365nm & 405nm). Next, the electrodes are metalized by spatter deposition (Bestec Berlin, Germany) of 200nm gold onto another patterned photoresist (AZ, MicroChemicals GmbH, Ulm, Germany), followed by a lift-off process in Dimethyl sulfoxide (DMSO), which removes the photoresist residuals. Then, an additional SU-8 layer is patterned into the micro-well structures to serve as a passivation layer that isolates the electrical current. Next, the implant is released from the Si wafer, using wet etching of the sacrificial layer. Finally, the implant is bio-functionalized by RGD oligopeptide serving as a bio-adhesion molecule. A detailed description of the process, the challenges, and how they were overcome are discussed in the Results section and the Supp. Material.

### Structural characterization of the device

Following the fabrication process, the device was structurally characterized using various methods, described next. An *Olympus BX51M microscope* (Tokyo, Japan), equipped with a DeltaPix Invenio 5SCIII microscope camera (Smorum, Denmark), was used to examine the implant’s overall structure and photoresist flow. A *profilometer* (Stylus Profilers, Bruker's Dektak®, MA, USA) was used to measure the sample cross section profile (1000nm, 60s). A *scanning electron microscopy* (E-SEM, Quanta FEG 250 by FEI, Hillsboro, Oregon, USA) and a *focused ion beam microscopy* (FIB, Helios 600, FEI Company, Hillsboro, Oregon, USA) were used to visualize the implant structure and to investigate the materials’ bond integration, specifically at the gold-SU8 interface. The acquired images served for process optimization to analyze the photoresists’ development and profile.

### Bio-functionalization

Of major interest to the field of electronic implants and neural prostheses is rendering the device biocompatible and enhancing the neuron-electrode proximity. To this end, the gold electrode surface was immersed in an aqueous solution of the biological adhesion motif RGD (0.1mM), followed by rinsing with DDW, resulting in a self-assembly monolayer (SAM) of RGD [30–32]. Briefly, the RGD-gold electrode coating was obtained through SAM by semi-covalent bonds forming spontaneously between the gold and the thiol group (SH) present in the RGD, which can be found at cysteine (C) amino acid [33–35]. As a starting point, the linear sequence CGGRGDSP (Adar Biotech, Herzliya, Israel) was used [30, 36, 37].

### Surface chemistry analysis

Investigation of the RGD molecule adhesion to the gold surface was conducted by *X-ray photoelectron spectroscopy* (XPS, Kratos Axis HS spectrometer, England) equipped with a monochromatic Al Kα X-ray source (photon energy 1486.6eV). Survey and high-resolution spectra were acquired at a pass energy of 80eV and 40eV, respectively. The source power was normally set to either 75W or 150W. The binding energies of all elements were recalibrated by setting the CC/CH component of the C 1s peak at 285eV. Quantitative surface chemical analysis was performed using high-resolution core-level spectra after the removal of the nonlinear Shirley background. The measurements were carried out under UHV conditions, at a base pressure of 5 × 10^–10^ torr (and not higher than 3 × 10^–9^ torr). Examinations were performed on coated mica glass disks (Electron Microscopy Sciences, Hatfield, PA, USA). The need for further surface functionalization stems from the fact that the SU-8 polymer repels cells due to its hydrophobic nature, induced by its epoxy groups. We therefore treated the devices using *dry etching plasma* (Dainer electronics, Pico, Germany) with various gases: O_2,_ N_2,_ or Ar (150W, for 3 min). This treatment is known to break the epoxy rings and form hydroxyl (R-OH) and carboxyl (-COOH) groups; consequently, this raises its surface energy and its wettability, leading to better biocompatibility [17, 38–40].

*Contact angle goniometer* (System OCA, model OCA20, Data Physics Instruments GmbH, Filderstadt, Germany) was used to assess the surface wettability, aiming to investigate the SU-8 bio-functionalization. Briefly, drops of 5μL of DDW were placed on the center of three SU-8 films, two of which were treated by N_2_ and O_2_ dry-etching plasma (150W, 3min), whereas the third served as a control. The measurements were performed at 25°C and with 55% moisture; Laplace-Young curve fitting was used to determine the static water contact angle values [41].

### In vitro characterization

#### Cell adhesion characterization

To investigate the cell adhesion with both the gold electrode and the SU-8 layer surfaces, two types of retinal-related cells were used, namely, the human retinal pigment epithelial cell line (ARPE) and rat photoreceptor precursors **(**rPRP**)**. ARPE cells were seeded on RGD-treated and untreated flat gold electrodes, in a medium containing DMEM (Biological Industries, Israel, Beit-Haemek, Ltd.) supplemented with serum (Gibco), L-glutamine (Biological Industries, Israel, Beit-Haemek, Ltd.) B27 and 100 µl/ml penicillin, 100µg/ml streptomycin, and 0.25µg/ml amphotericin (Biological Industries, Israel, 03–033-1B). The rPRPs were dissociated from P1 SD rats and were seeded on various SU-8-treated surfaces (N_2_, O_2_ dry-etching plasma and control). Both cells were incubated at 37°C with 5% CO_2_. The rPRP cells were imaged for cell survival at days 1 and 4 post-seeding.

The ARPE cells were fixated at day 3 post-seeding, after which the samples were prepared for SEM/FIB imaging according to our previous report [42]. Briefly, samples were washed using PBS and were primarily fixed with Karnovsky Fixative buffer (#15720, Electron Microscopy Sciences). Next, samples were stained for 1h in 1% osmium and finally, samples were dehydrated in increasing concentrations of ethanol and then left to dry overnight. The dried samples were then coated with a 20nm gold layer (Quorum Q150T ES), at which point the samples are ready for SEM imaging (E-SEM 326, Quanta FEG 250 by FEI).

To quantify the effect of electrode coating on cellular adhesion, acquired SEM images were analyzed using ImageJ. As a measure of cellular adhesion, we defined the adherence ratio as the percentage of electrode area occupied by cells (for all electrodes in the FOV), divided by the percentage of the non-electrode area occupied by cells in the corresponding field of view. A ratio higher than one suggests that the cells were attracted to the electrodes.

#### Ex-vivo retinal electrical stimulation

Since the ultimate goal is to utilize this implant as a subretinal prosthesis, we explored the feasibility of our high-resolution device to serve for the subretinal stimulation of an isolated retina. To this end, we used two types of devices: in the first, the implant prototype was adapted in such a way that alternating rows were short-circuited to enable a simple electrical stimulation. In the second, the implant was composed of 60 high-density electrodes (an electrode pitch of 50µm), which could be addressed individually. Both prototypes were interfaced with a multi-electrode array current injector (MEA 2001, Multi-Channel System, Germany). Either biphasic cathodic first or cathodic pulses, 0.1ms-25ms, 10µA-100µA, were delivered at a rate of 0.2Hz. We utilized retinas from transgenic animals that incorporate the genetic calcium indicator GCaMP6, allowing for the optical monitoring of retinal ganglion cell activity in response to subretinal electrical stimulation. The retina, isolated in an oxygenated Ringer’s medium, is mounted with the photoreceptors face down on the implant and filled with oxygenated Ringer’s medium (see the Supplementary Material). The electrically induced RGC responses were visualized by an *upright microscope* (Slicescope 6000, Scientifica, Uckfield, UK) equipped with a *CCD camera* (EXI-Blue QIMAGING) and a filter set (EX 488nm/ EM 525nm) to allow for fluorescent image acquisition at 10 frames per second. The activation threshold was calculated from the change in the fluorescence signal from the baseline (as an indicator of RGC activity) using a custom-written software as was previously described by our group [43] (see the Supp Material).

#### In-vivo and histological studies of the implant integration in the rat retina

All animal experiments were approved by the Bar-Ilan University Ethics Committee for Animal Research and were conducted in accordance with the Association for Research in Vision and Ophthalmology Statement for the use of Animals in Ophthalmic and Vision Research. Long Evans pigmented rats (12 weeks old) were anesthetized with an IM injection of Xylazine (6 mg/kg), ketamine (100 mg/kg), and atropine (0.06 mg) with the addition of a topical application of Lidocain 2%; the device was implanted in the subretinal space of the rats and was monitored for over a month using a method previously reported by our group [44]. Briefly, Optical coherence tomography (OCT) and fundus camera (Micron IV, Phoenix Research Laboratories, Pleasanton, CA, USA) imaging were utilized for investigating the anatomical integration of the implant with the host retina, similar to our previous reports [44–48].

Following imaging, the implanted rats were euthanized, and their eyes were incubated with 4% paraformaldehyde for 24h, after which the eyes were rinsed using PBS and were flat mounted. The flat mount tissue was nucleus stained using Hoechst (#14,533, Sigma- Aldrich) and imaged by a *confocal microscope* (Leica TCS SP8).

Alternatively, to investigate the integration with the host retina, *cryosectioning* was performed. To this end, the tissue was incubated with increasing percentages of sucrose (Millipore, 573,113-1kg), (5, 15, and 30%) at room temperature for 15min each, then incubated with PBS containing 30% sucrose for 24h at 4°C. At the end of the process, the tissue was frozen in a stable orientation in OCT medium (Tissue-Plus, OCT compound embedding matrix Tissue-Tek Scigen), and then cut into 10µm-thick slices with a cryostat (CM1800 LEICA). The retinal cryosections were stained with the retinal bipolar primary antibody PKC alpha (#P4334, Sigma-Aldrich).

## Results

During the optimization of the complex fabrication process, several challenges that affected the implant quality and feature resolution were overcome. The main challenges include the optimization of UV exposure for achieving a high aspect ratio of SU-8 wells, the formation of dense gold microelectrode arrays with sharp edges and with strong bonding to the SU-8 surface, avoiding thermal stress development within the SU-8, avoiding the creation of "streaming lines" due to a multilayer lithography process, implant release, and bio-functionalizing.

### Optimization of the UV exposure in a high aspect ratio device

**Fig. 3:**
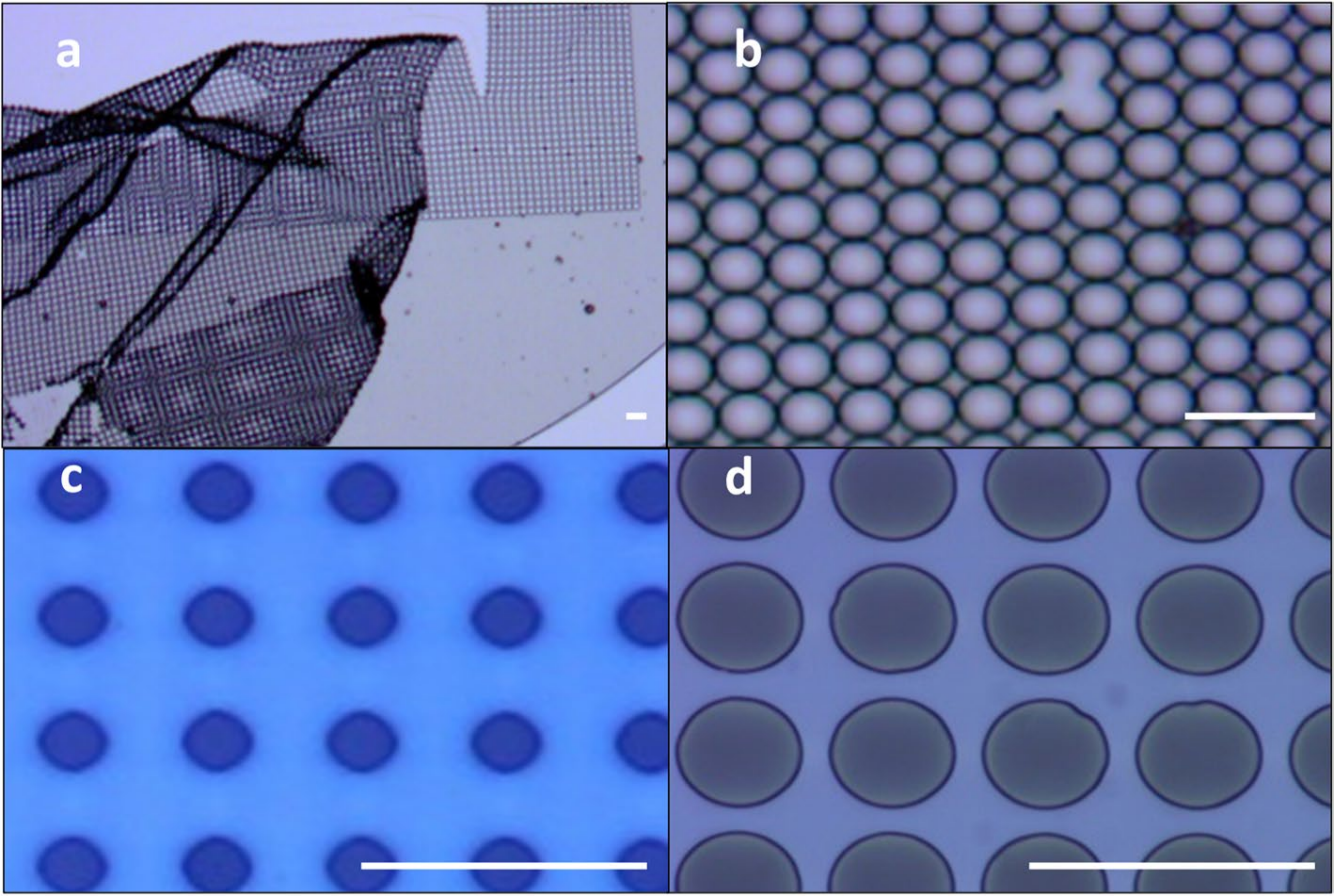
UV dose effect on the SU-8 patterning. **a**) An extreme underexposure dose leading to low mechanical strength and rupturing of the film. **b**) Underexposure dose leading to the expansion (22 µm) of the micro-wells and the fusion of adjacent wells. **c**) Extreme overexposure, leading to the closing and reduced diameter of the micro-wells. **d**) Optimal UV dose resulting in perfectly circularly shaped micro-wells of the desired diameter (20 µm). Scale bar - 50 µm in all figures

Optimization of UV exposure during a complex lithography process of a 3D high-aspect ratio device is affected by numerous factors such as the type of photoresist (positive or negative), geometry, the reflections due to the presence of metal electrodes, and more [29, 49] (see the Supp. Material). The exposure dose ranges from underexposure to overexposure; thus, in order to avoid undesired structural defects, the optimal exposure dose needs to be determined. To this end, for each photoresist, substrate and thickness manual optimization was carefully performed using the designated hashtag #, as is described in the Supp. Material and can be seen in Fig. 3 and Figs. S1 and S2. Using these optimization steps, we were able to achieve perfectly circular shaped micro-wells in the desired dimensions for the negative SU-8 photoresist (Fig. 3d) and the positive AZ photoresist (Fig. Supp. S1).

### Dense gold microelectrode on SU-8: strong attachment, high resolution, and sharp profile electrodes

The electrode fabrication process involves thin film metallization of gold by spattering onto the patterned AZ photoresist, followed by a lift-off process that removes the undesired metal (gold) at the unexposed photoresist areas. Two main factors affect this method and limit a successful fabrication of sharp, flat, and clear-cut metal electrode edges: the strength of the bond between the deposited metal (gold) onto the surface (SU-8) and the patterned photoresist profile.

The deposited gold adheres weakly to the SU-8 polymeric surface due to the gold’s inertness and its low surface energy in conjunction with the poor wettability (hydrophobicity) of the SU-8 [40], resulting in electrode detachment and rupture during the lift-off process [49] (Fig. S3). To increase the electrodes’ adherence to the SU-8 surface, we applied several methods aiming to increase the SU-8 surface energy: dry plasma etching (O_2,_ Ar, or N_2_) treatments for various times and powers (1min to 5min and 100W to 300W, respectively) were applied onto the SU-8 before metallization to modify the SU-8 surface [17, 40] by breaking the epoxy rings, resulting in hydroxyl and carboxyl edge groups [17, 50, 51]. The various dry etch plasmas’ impact on the SU-8 hydrophilicity was evaluated by contact angle measurements (Supp. Fig. S9a-c). In addition, argon (Ar) ion milling was applied prior to the sputter deposition process, in the same chamber, to further increase the SU-8 surface energy. Moreover, we investigated the use of the metal adhesion layers (Ti and Cr) at various thicknesses (5nm-20nm) with the aim of bridging the hydrophobic SU-8 nature and the gold’s inertness (data not shown). Following the optimization process, we concluded that O_2_ plasma (150W, 5min), combined with (Ar) ion-milling (10s) and the use of a Cr adhesion layer resulted in the best adhesion.

**Fig. 4:**
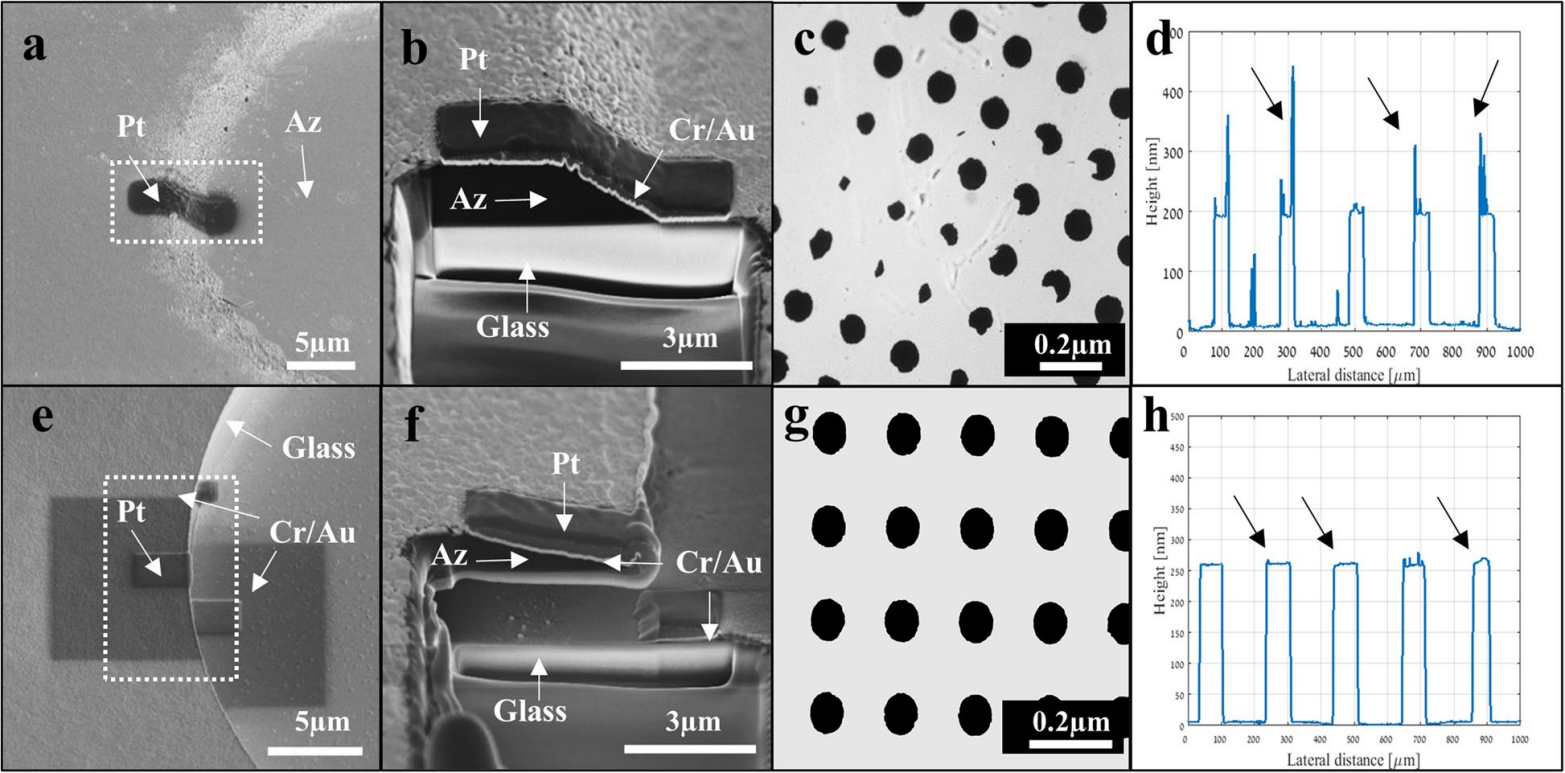
Optimization of gold microelectrode fabrication. **a**-**d**) A ruptured electrode with an "ear pattern" profile fabricated using the conventional AZ monolayer lift-off process and developed for 1 min. **a**) Top view SEM imaging (the Pt line is deposited for FIB/SEM and is not part of the fabrication process). **b**) FIB/SEM zoom in the cross section of the area demarcated with a rectangle revealing the continuity of the photoresist. **c**) Gold electrodes (80 µm diameter, 200 nm height) are broken and disconnected from the substrate. **d**) The electrodes’ edge cross section profile showing the “ear pattern” edge (arrows). **e**–**h**) An intact electrode with a sharp profile fabricated using the bi-layer lift-off process (AZ and LOR) developed for 1 min, 5 min curing at 185 °C and another development phase of 1 min. **e**–**f**) SEM imaging of the positive photoresist profile optimization process of a Bi-layer lift-off with LOR10B. **e**) An SEM image of the top view of a photoresist developed for 1 min. **f**) An FIB/SEM cross section and a zoom-in on the white rectangular area demarcated in) (**e**) (where the LOR10B was developed for 1 min, followed by an additional AZ curing step (120ºC). The photoresist has the desired undercut configuration, which is denoted by *. **g**) Intact gold electrodes (80 µm diameter, 200 nm height) with good attachment to the SU-8 surface. **h**) An electrode edge cross section profile showing the sharp edge (arrows)

The second challenge for the successful patterning of metal electrodes using a lift-off process is to achieve a proper patterned photoresist profile. Using a negative photoresist for deposition has the advantage of creating the desired trapezii profile [52], which has the advantage of creating clean and sharp electrode edges during the lift-off process, however it is limited in resolution and expensive. On the other hand, a positive photoresist is usually used for high-resolution features, but has the drawback of creating a typical concave (bowl-like shape) profile (Fig. 4a-b), which results in continuity of the metal deposition and increases its lateral surface tension [53] (Supp. Fig. S4), eventually causing tears in the gold electrode during the lift-off process (Fig. 4c); this leaves the so-called “ear-pattern” gold residuals at the electrode edge (Fig. 4d). We opted to use the positive resist and to achieve the desired "negative-like" profile by using a bi-layer lift-off process (Fig. 4e-h) [54–56]. The bi-layer process utilizes an additional layer of a fast-developing resist (e.g., PMGI, LOR) under the positive photoresist. This layer dissolves faster than the patterned photoresist during the photoresist development after UV exposure, therefore resulting in an "undercut" profile and thus efficiently separating the desired regions from the undesired metal regions as in the negative "trapezii" shape. In order to achieve the desired undercut profile, various materials with different thicknesses and dissolution rates (such as LOR10B, PMGI sf3, and PMGI sf6) were investigated. Briefly, by implementing a second cycle of a curing step at a temperature higher than the photoresist (i.e., AZ) glass transition temperature (T_g_) and lower than that of the dissolved layer (120°C for 1 min), as proposed by Wilson et al. (2015) [56], we could control the dissolution rate and the desired undercut profile. Figure 4e-h. presents the results of the optimal bi-layer lift-off process with the additional curing step. The desired discontinuity between the gold layer and the photoresist can be seen in the FIB/SEM images (Fig. 4e-f); this leads to a complete intact circularly shaped electrode (Fig. 4g) with a clear-cut sharp profile (Fig. 4h).

### Avoiding thermal stress

During the curing steps a thermal stress is prone to develop in the SU-8 due to the mismatch between the thermal expansion coefficient (CTE) of the Si wafer (2.6ppmC^−1^) [57] and the SU-8 (52ppmC^−1^) [58], resulting in cracks within S-8. We solved this issue by two different approaches. First, we iteratively optimized the heating and cooling gradient following curing (7°C min^−1^). More importantly, we added an intermediate layer of Crumuim-Nickel (13.3 ppmC^−1^) [59] between SU-8 and the gold layers (see Figs. 1 and 2). Indeed, these steps significantly eliminated the thermal stress, as shown in Fig. S5 in the Supp. Material; the optimized protocol is described in the Supp. Material.

### Multilayer lithography process: streamlines and mechanical stress

While fabricating a 3D multilayer device via a layer-by-layer photolithography process, the cumulative effects of each of the previous steps affect the proceeding ones. One such challenge is the patterned substrate topography, which results in a "streamline" effect of the current photoresist layer, which prevents uniform coating through standard spin coating. Briefly, in order to overcome this challenge, we optimized the spinning protocol to include several steps, which led to a homogeneous uniform coating of the various photoresist layers. The method we used to overcome this is described in the Supp. Material and Fig. S6.

### Implant release

Since SU-8 is an epoxy polymer, it tends to adhere to surfaces during polymerization, preventing the release of the device from most wafer substrates. In some processes, a striping technique using the commercially available Omnicoat has been introduced [15]; however, it could not be used in our complex multiple step process because of its interference with the following steps. Therefore, we adopted a “sacrificial” layer approach [60], whereby a sacrificial metal layer is deposited onto the substrate wafer below the SU-8 and is wet-etched by acid at the conclusion of the fabrication process. However, since the same acid can also etch the gold electrode or the Cr/Ti adhesion layer, the process had to be optimized to prevent electrode detachment. Briefly, we found that the best release can be achieved using a 200nm Ni or Cu as a sacrificial layer that is wet etched by 21% NHO_3_ overnight, similar to Feiner et.al [3] at the end of the process. This, however, results in a yellowish SU-8, which was further prevented using ammonium peroxydisulfate salt (Merck, Germany) 10%v/v instead of the acid. Figure S7 in the Supp. Material presents the electrode adhesion integrity before and after the optimization.

### Bio-functionalization of the implant by adhesion molecules and surface treatment

**Fig. 5:**
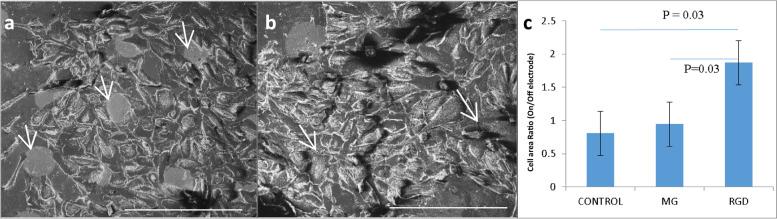
Effect of various coatings on cell adhesion to gold electrodes. **a**-**b**) SEM image of ARPE cells seeded on gold electrodes (40 µm in diameter, white arrows), used to quantify the effect of various coatings on cell adhesion and the preference of gold electrodes without RGD (**a**) and with RGD (**b**); the scale bar = 200 µm. It can clearly be seen that although the cells tend to be repelled by bare gold electrodes (**a**), the cells tend to adhere to RGD-coated gold electrodes (**b**). **c**) The obtained adhesion of cells to gold electrodes for the various coatings defined as the prevention of the electrode area occupied by cells divided by the percentage of the area surrounding the electrodes occupied by cells. Values larger than the one obtained for the RGD coating reveal the cell’s preference for electrodes following this treatment, compared to the control

Since the bio-functionalization of the implant plays a critical role in enhancing cell-electrode coupling, we investigated the effect of the functionalization of gold electrodes with a linear RGD pentapeptide and the effect of dry etch plasma on cell attraction and adhesion to the electrode surface. RGD is one of the extracellular matrix (ECM) cell recognition motifs that connect cellular integrins; thus, incorporating this molecule in a device increases cell adhesion to the surface [61, 62]. The presence of the RGD on the electrodes was verified by XPS chemistry (Fig. Supp. S8). The effect of RGD functionalization on the electrode cell interface was studied by seeding retinal relevant cells (ARPE) on electrodes functionalized with RGD and comparing the results to cells cultured on electrodes coated with Matrigel or using uncoated gold as the control (Fig. 5). It can be seen that the adherence ratio (see the Methods) was significantly larger when the electrode was coated with RGD, compared with Matrigel and the control, suggesting the facilitation of cell adherence to the functionalized gold electrodes (*p* < 0.05, unpaired Students t-test, Fig. 5c).

**Fig. 6:**
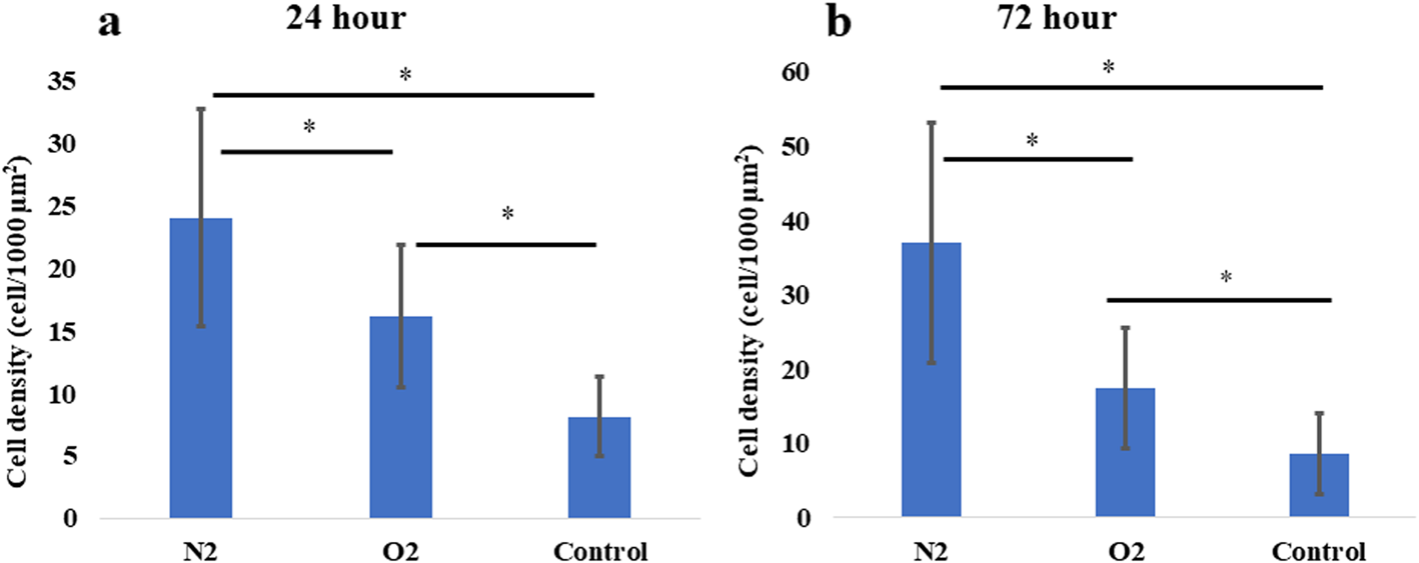
Effect of plasma surface treatment on SU-8 biocompatibility. Rat PRP cell density on N_2_ or O_2_ plasma-treated cured SU-8 (150W, 3 min) compared to not treated cured SU8. 24 h (**a**) and 72 h (**b**) post seeding. * Denotes *p* < 0.05

In addition to electrode functionalization, we addressed the SU-8 surface, which is known to repel cells, because of the hydrophobic epoxy surface structures. Aiming to further bio-functionalize the implant, we used dry plasma etching (150W, 3min of O_2_ or N_2_), which breaks the epoxy rings while creating reactive group chains; thus, oxidizing the surface and increases its hydrophilicity [17]. The plasma treatment effect on the surface wettability was assessed by contact angle measurements (Supp. Fig. S9a-c), which showed increased wettability in both the O_2_ and N_2_ plasma treatments (a higher contact angle compared with untreated SU-8). Twenty four hours post seeding rPRP cells density was significantly higher on N_2_ plasma treated SU-8 compared to O_2_ plasma (24.0 ± 8.6 cells/1000µm^2^ vs 16.1 ± 5.7, average ± STDEV, p = 0.02, for N_2_ plasma and O_2_ plasma, respectively) and compared to no treatment (13.0 ± 16.2 cells/1000µm^2^ vs 17.4 ± 8.0 cells/1000µm^2^, P = 0.002, for no treatment and N_2_ plasma, respectively). Similar results were found for 72h post seeding, as is shown in Fig. 6b (and Supp S9d-i).

### Final fabrication process

The final fabrication process is depicted in Fig. 2 and is detailed in the Supp. Material.

### Characterization of the Retinal Implant

**Fig. 7:**
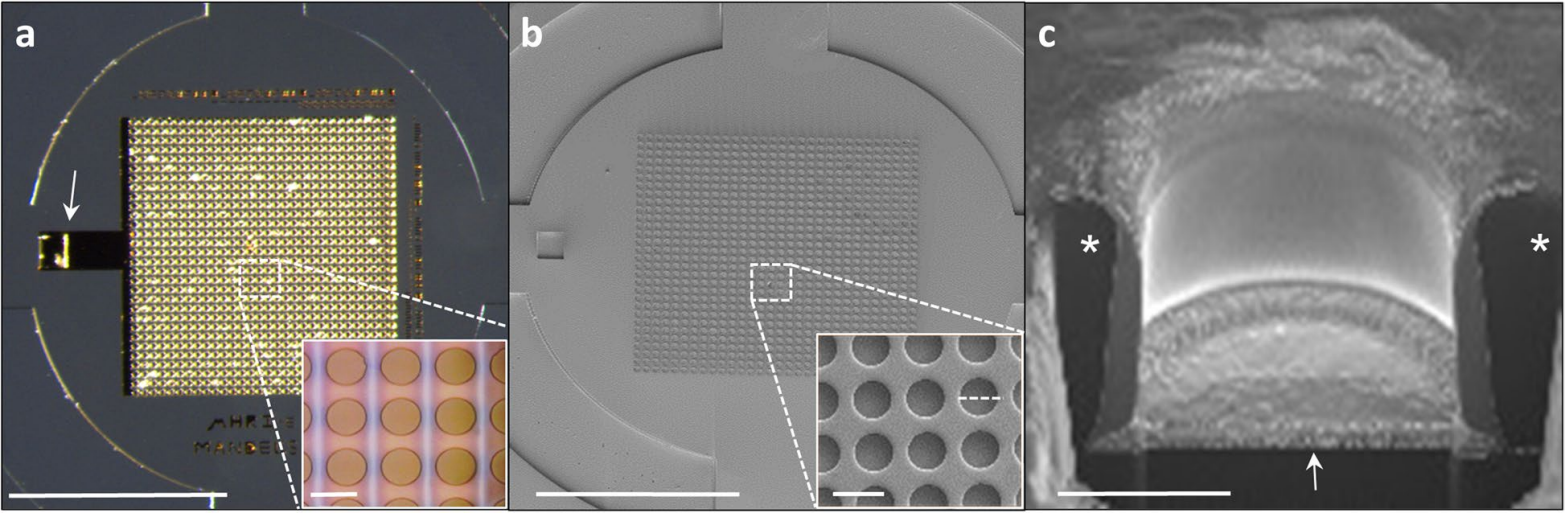
Images of a completed retinal implant (1mm in diameter) with a gold electrode array. **a**) Color image; the top view of a full SU-8-gold retinal implant; scale bar = 0.5mm. The insert is a zoom-in on the area demarcated by the rectangle (scale bar = 20µm). **b**) SEM images; the top view of the implant as in a); scale bar = 0.5mm. In the insert, a zoom-in on the area demarcated by the rectangular; scale bar = 20 µm. **c**) A FIB/SEM cross section image of the 3D well-like structure encapsulating the electrode; scale bar = 10µm. The black pillars are the SU-8 micro-wells walls (*) and the gold electrode (the white arrow). Scale bar = 10µm

Figure 7a depicts a bright-field microscopy image of a completed 1mm diameter implant with a dense circular micro-well electrode array. Further characterization of the implant at higher resolution using SEM (in Fig. 7b) revealed the dense micro-well-like structures and the electrodes with clear-cut features (20μm in diameter, 23μm pitch to pitch). Cross sections obtained through FIB/SEM (Fig. 7c) depict a single micro-well with a gold electrode at the bottom (arrow) with good structural integrity of the various implant components.

### Ex-vivo subretinal stimulation

**Fig. 8:**
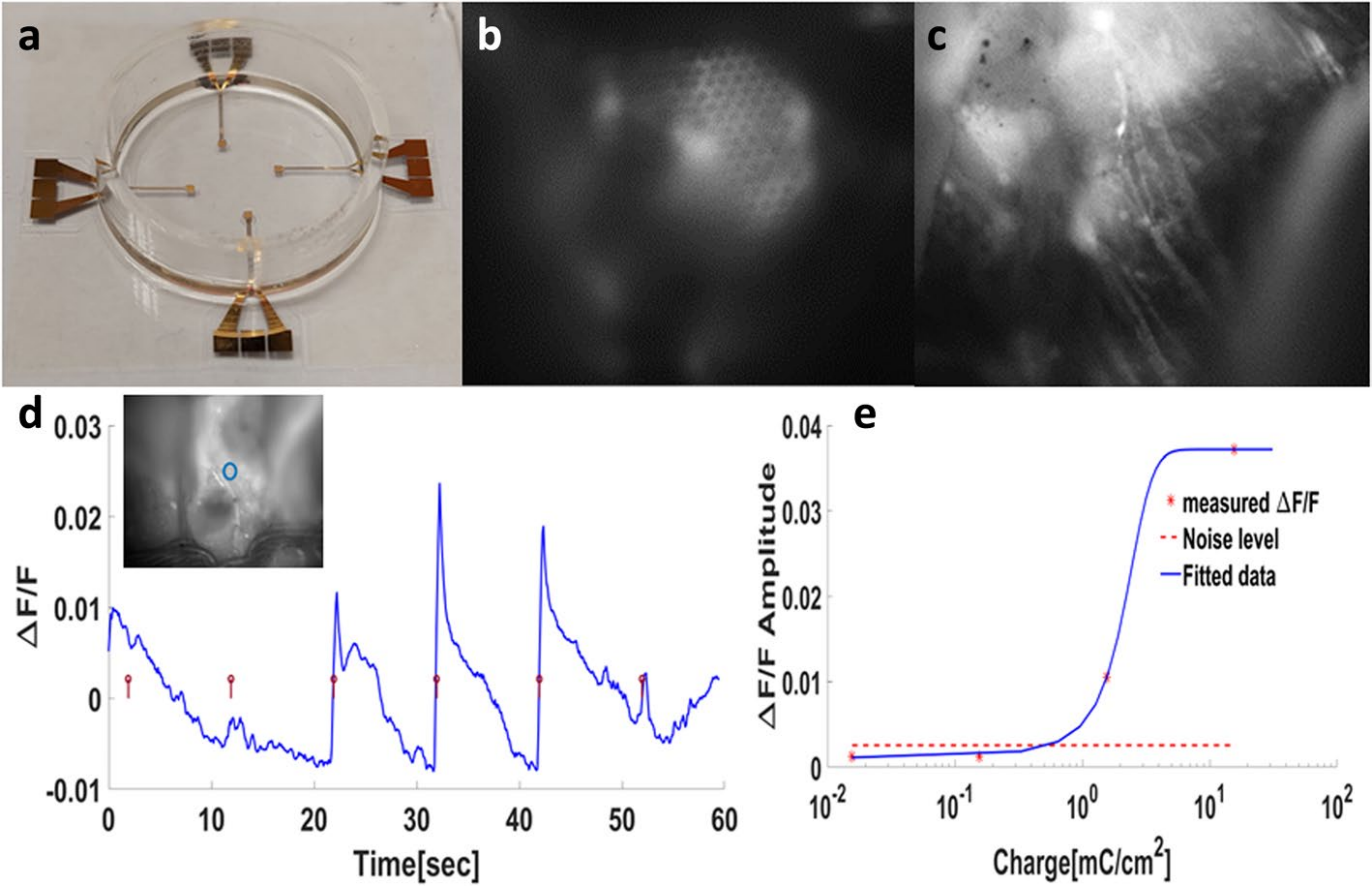
Ex-vivo retinal stimulation proof of concept. **a**) The conceptual implant is placed in a dish with Ringer’s medium. **b**) A fluorescence image of the isolated retina harvested from transgenic GCaMP6f-Thy1 rats mounted on the implant. Arrows point to the micro-wells. **c**) The same as in b with the focal plane adjusted to show the fluorescent RGCs and axons (arrows). **d**) Average fluorescence change in response to electrical stimulation with increasing current density. **e**) Average fluorescence change in response to electrical stimulation with increasing charge density per phase, indicating an activation threshold below 0.156 $\frac{mC}{{cm}^{2}phase}.$

The investigation of the implant feasibility to serve as a subretinal neurostimulator is presented in Fig. 8. As described above, the implant was fabricated on glass and mounted in a multi-electrode stimulation system (Multichannel Systems, Inc.) (Fig. 8a). Transgenic rat retinas expressing the calcium indicator GCaMP6 in their RGC were placed on the implant RGC facing up (Fig. 8b and c), and retinal ganglion cell responses to the implant electrical stimulation were observed through calcium imaging experiments. Figure 8d shows a robust significant repetitive fluorescence signal change indicating the successful subretinal stimulation of the isolated retina. Increasing the stimulus charge resulted in the expected sigmoidal increase in the RGC responses (Fig. 8e). Experiments investigating the activation charge threshold revealed the activation thresholds of 0.156mC/cm^2^ per phase, comparable to values reported in the literature [63–66] (Fig. 8d). To validate the nature of the observed activity and to rule out potential artifacts, we added the voltage-gated calcium channel blocker Verapamil (at a concentration of 200 µM). Upon the addition of this blocker, all activity was diminished and was successfully restored upon washout (Supp. Fig. S10), further validating the physiological nature of the observed fluorescence change. Furthermore, using a high-resolution ex-vivo prototype of the implant, we could stimulate localized area of excised retinas (Supp Fig. S11a,b); the activation threshold showed a characteristic strength-duration function (Supp Fig. S11c).

### In-vivo characterization

**Fig. 9:**
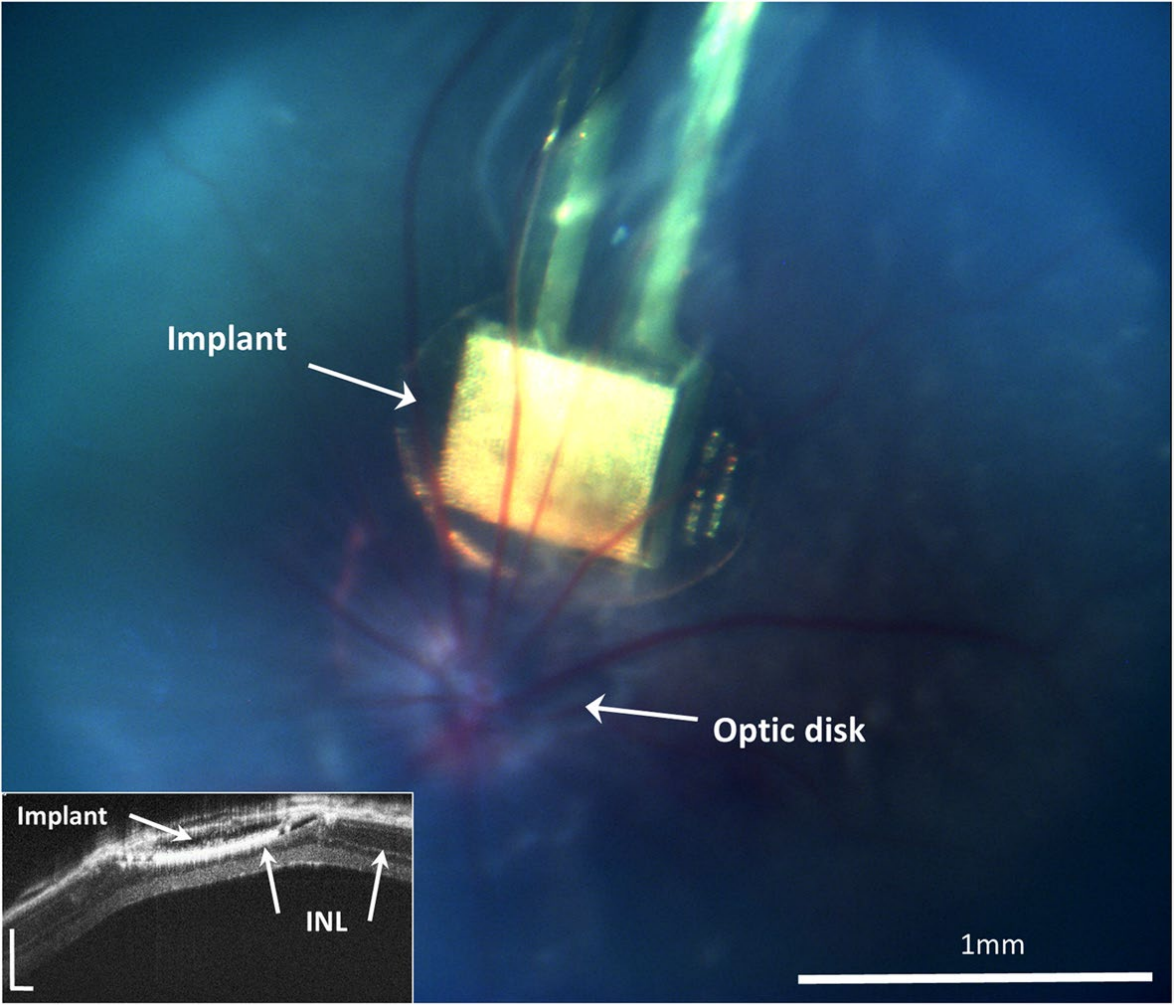
Integration of the implant in the sub-retinal space. Fundus image of the implanted device (white arrow) demonstrates the good placement near the optic disk; scale bar = 1mm. In the inset, an optical coherence tomography cross section reveals good integration of the implant in the sub-retinal space under the inner nuclear layer (INL), ONL – Outer Nuclear Layer; scale bar = 200µm

**Fig. 10:**
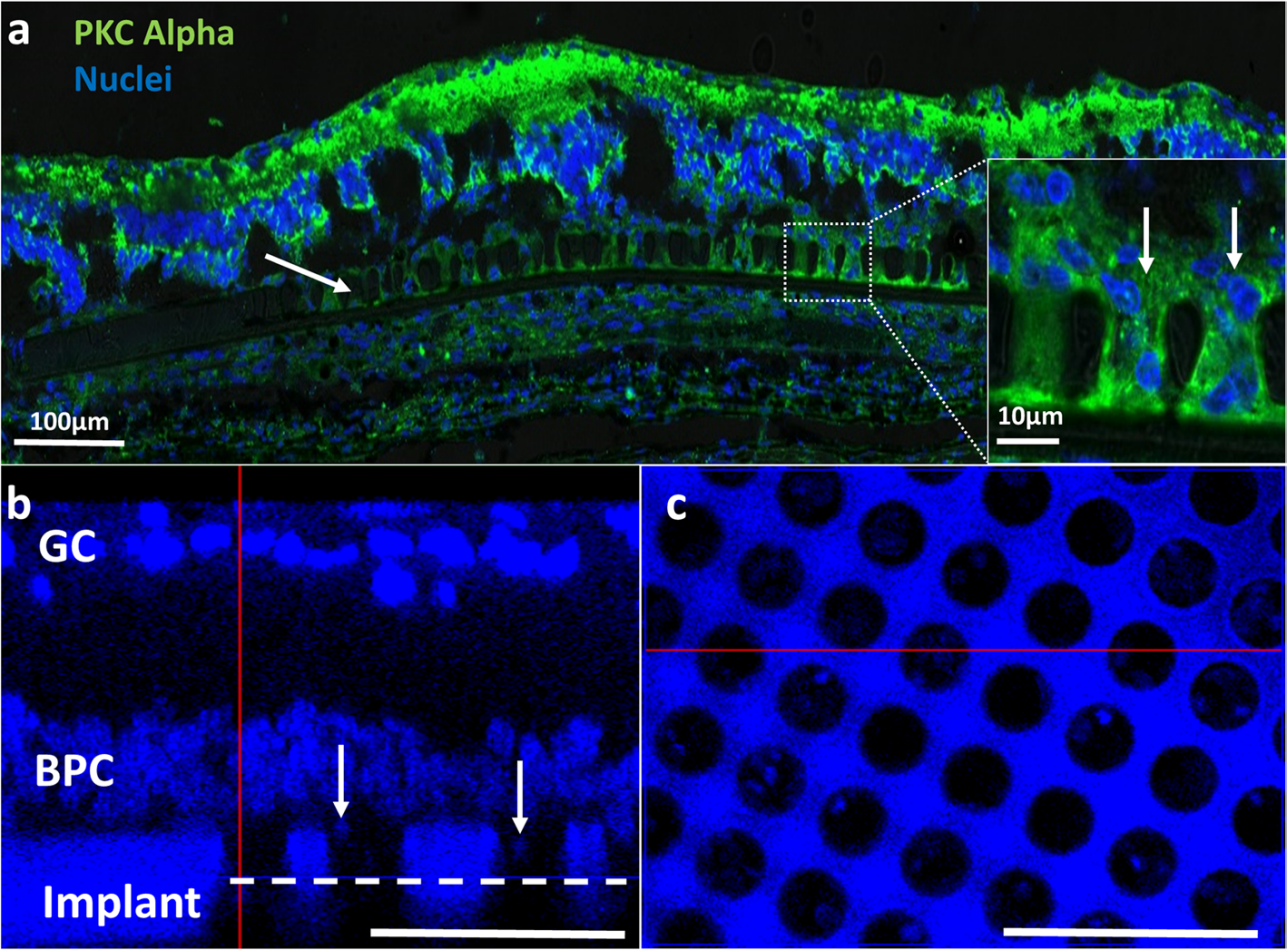
Histology of a flat mount retina implanted with the retinal device. **a**) Confocal imaging of the implanted retinal device (arrow) showing good anatomical integration in the subretinal space; scale bar = 100µm. The insert shows a high magnification of the micro-wells in the demarcated area showing proximity between the electrodes and bipolar cells entering the micro-wells; scale bar = 100µm. **b**) A cross section of the implanted retina showing the proximity between the implant and the BPC layer with some of the cells migrating towards the micro-wells (arrows). **c**) A cross-section at the micro-wells’ mid-height (reference point: the white dashed line in b) revealing the presence of bipolar cells within the micro-wells. Scale bar = 50µm. GC—ganglion cells, BPC—bipolar cells. Green—PKCα, blue—Nuclei

To investigate the integration of the device within the retina, it was implanted in the subretinal space of Long Evans rats. Fundus camera imaging and optical coherence tomography (OCT) were performed at 30-days following implantation. The images (Fig. 9) revealed the good anatomical integration of the implant in the sub-retinal space. OCT imaging further highlighted the good proximity between the device and the inner nuclear layer (INL), where the target cells (bipolar cells) are located (Fig. 9, insert). The animal was then euthanized, and the whole mount eye was fixated, treated for nuclear staining (Hoechst), and the bipolar cell marker PKC alpha, and then imaged by confocal microscopy. As shown in Fig. 10, the implant is located in the desired location of the subretinal space (arrow), with some bipolar cells migrating into the micro-wells (insert), as was previously reported [67].

**Fig. 11:**
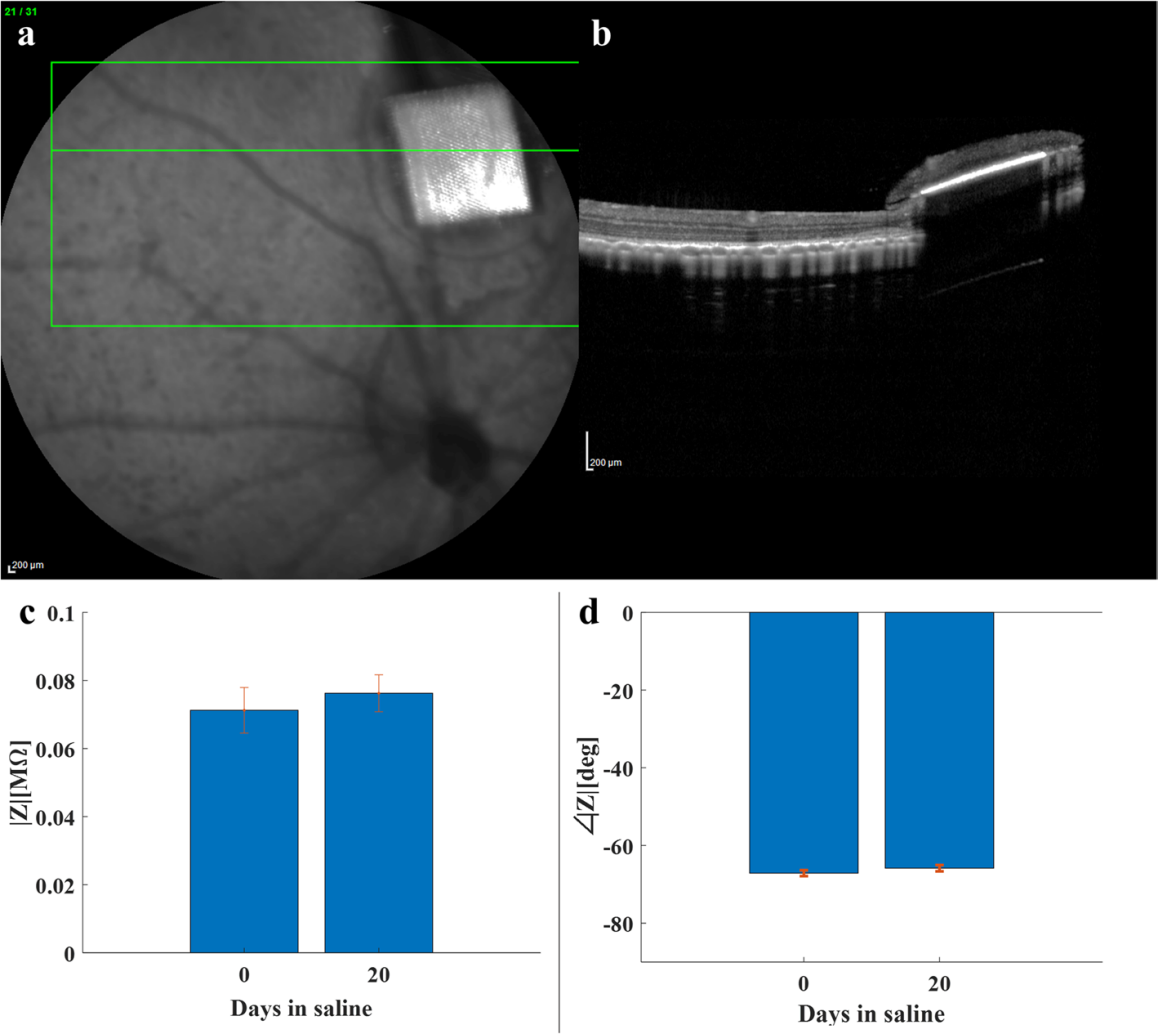
Implant Stability. **a**) OCT imaging performed 3 months following transplantation, revealing the structural integrity of the implant and its correct location in the subretinal space. **b**) The implant impedance at 1kHz was measured before and after a 20-day incubation, showing no significant changes in the electrodes’ impedance (*p* > 0.2)

Four animals were followed up for up to 3 months and repeated OCT imaging showed the long-term implant structural stability (Supp Fig. 11a, b). Furthermore, long-term incubation of the implant in saline 0.9% at 37°C showed no significant effect on the electrode impedance (*n* = 7), as was measured at 1kHz using the NanoZ (Multi Channel System) (*p* > 0.1) (Fig. 11c, d).

**Fig. 12:**
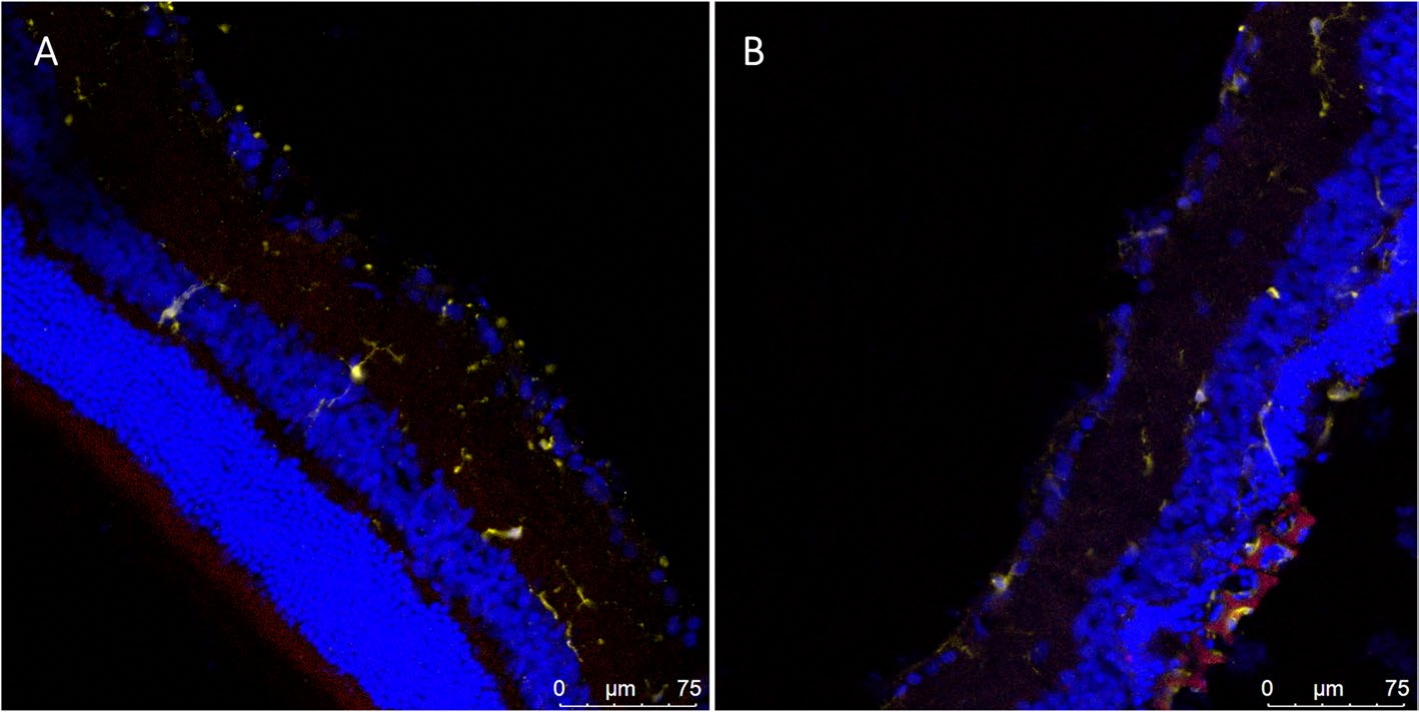
IBA-1 staining—Cryosections of a control retina (**a**) and an implanted retina (**b**) stained for stained for Hoechst (blue), IBA-1 antibody (yellow) and Rhodamine B (red), which visualized the nuclei in the various retinal layers, microglia and microphages, and the implant respectively showing no difference in the immune system response

To address the important issue of immune cell response, we performed the commonly used immune-cell antibody staining Iba-1 (see Supp. Material for the methods), which stains for microglia and microphages in the retina. We found no differences in the immune response of the implanted retina compared to control eyes (17.0 ± 3.2 vs. 19.8 ± 3.6, average ± STD, p = 0.18 per 500µm retinal slide, for control and implanted eyes, respectively (Fig. 12a-b).

Moreover, using proTUNEL staining, the implant was found to be biocompatible, as inferred by the similar number of viable ARPE-19 cells following culturing on the implant, compared to those cultured on the control surfaces (Supp Fig. S12).

### Discussion and future directions

This work involved the fabrication of a conceptual 1mm circularly shaped complex 3D implant aimed at neural stimulation of the retina. The implant consists of gold micro-electrodes located at the bottom of micro-well structures, with a high aspect ratio and is fabricated using an SU-8—metal—SU-8 configuration. This 3D microwell or “honeycomb” geometry was suggested to enhance cell-electrode coupling by vertically align the electric field and thus decouple the field penetration depth from the pixel width [67].

The optimal fabrication process consisted of three sequential cycles of photolithography formed on a silicon wafer coated with a thin layer of nickel, which was used as a sacrificial layer, followed by spatter deposition of chromium-gold metallization for electrodes patterned by a bi-layer lift-off process, implant release, and RGD bio-functionalization. Although this conceptual implant lacks a current source for neural stimulation (photovoltaic, wired or inductive), it was used for the detailed layer-by-layer optimization of the complex fabrication process, and thus can serve as a guide for fabricating similar devices.

The first challenge was overcoming the thermal stress, which develops in the SU-8 volume during the process, when applied on a silicon wafer. Similarly, to what Guo et al. [68] reported, we concluded that to overcome the effect arising from the different CTE values of the SU8 and the Si substrate, a slow and gradual rise in temperature (7°C min^−1^) is required, in addition to the use of high-quality masks. Moreover, an additional intermediate layer with an intermediate CTE value further reduced the thermal stress, similar to Abgrall [15]. In our work, the nickel layer (which has a CTE value of 13K^−1^) served both as a sacrificial and a thermal intermediate layer. Indeed, taking these measures resulted in a reduction of the thermal stress and prevented cracks in the SU-8 layer.

The second challenge we addressed during this process was the formation of the so-called “stream-lines”, which occur during fabrication of a multilayer photolithography process, due to the multilevel effects of each of the previous steps on the proceeding ones. This challenge was resolved by adopting a uniform coating technique, as described above. Next, we tackled with the optimization of the UV exposure dose required for the patterning of the photoresist. The main parameters determining the UV exposure dose are the photoresist height and the substrate reflectance, which create standing waves on its surface, preventing the proper exposure of the resist on its interface [49]. Although this can be partially solved by use of anti-reflecting coatings, the common materials used for this purpose fall short of resolving this issue because they lack sufficient mechanical and chemical properties necessary for integrating permanently in the device and thus become a potential failure point. Thus, we resorted to a step-by-step manual optimization of the dose through the use of fine-resolution marks.

Another major challenge was the poor attachment between the gold electrodes and SU-8 [69]. This obstacle was overcome by adding the steps of surface treatment (O_2_ plasma and Ar ion milling) to the SU-8 substrate after curing, and by adding an adhesion layer of chromium, which all improved the gold adhesion. More importantly, the electrode integrity was further significantly improved by generating an undercut, a “trapezii”-like, patterned photoresist profile by applying a bi-layer lift-off process, where control over the dissolution rate of the faster developed material (LOR) was achieved by an additional curing step to a temperature that is higher than the T_g_ value of the photoresist (i.e., AZ) and lower than that of the LOR itself.

Implant release from the wafer was achieved by using a sacrificial layer approach. Since the commonly used Omnicoat (MCC, USA) could not be used in our process, because of the technical constraints described above, we used a metal sacrificial layer. However, this approach, which utilizes wet etching, had to be optimized for preserving the metal electrodes and adhesion layers by optimizing the proper material combination and adjusting the height contrast between the electrode adhesion layer and the sacrificial layer.

Finally, in order to increase the bio-functionalization of the electrodes to attract the retinal neurons and increase the neuron-electrode coupling, the gold electrodes were functionalized by an RGD oligopeptide, a motif of the extracellular peptide fibronectin, which connects to the cell integrin peptides [69]. This RGD monolayer forms spontaneously on the gold surface via a thiol group (SH) when immersing the gold electrodes in aqueous RGD solution overnight at room temperature. This bio-functionalization was performed at the conclusion of the fabrication process and was shown to increase the attraction of neurons to the electrodes in vitro.

The feasibility of using the final implant was demonstrated by successful localized ex-vivo retinal stimulation and was further explored by implanting the device in the sub-retinal space of rats. The later experiments revealed a good anatomical integration of the implant, which was demonstrated by OCT and by histology showing close proximity between the bipolar cells and the electrodes. Our findings additionally demonstrate the migration of bipolar cells into the micro-wells, consistent with a previous study [68], which further confirms the successful integration of the implant into the retinal tissue. Moreover, our results indicate the long-term durability of the implant in both in-vitro and in-vivo settings, with no notable indications of a substantial immune reaction.

## Conclusions

In conclusion, we described here the optimization of a micro-fabrication process of a conceptual implant. We designed a conceptual sub-retinal prosthesis implant composed of a high-resolution electrode array with micro-wells (20μm in diameter) at a high aspect ratio. The various challenges faced throughout the fabrication process of the 3D implant and the approaches pursued for resolving the issue are described in detail. Among the challenges include proper photoresist coating, overcoming thermal stress, improving the adhesion between the gold and the SU-8 polymer, obtaining a sharp electrode profile by a bi-layer lift-off process, and the implant release. Furthermore, the cell growth onto the implant and the electrode was improved by bio-adhesion molecule coating and dry-plasma surface treatment. As a first proof of concept, in-vivo investigations, which examined the implant integration in rat host retina for over 3 months, revealed a good anatomical integration with the host retina, along with no significant retinal inflammatory response. The device enabled the localized electrical retinal stimulation, as shown by ex-vivo studies. The results of this optimization process can be applied in the fabrication and development of other neural prosthetic implants aimed at restoration of neural function or for other bio micro-devices.

### Supplementary Information

**Additional file 1:** **S1. **UV dose effect on AZ positive photoresist patterning. a) Photoresist residues inside the AZ pattern as the result of underexposure. b) A clean AZ pattern following a sufficient exposure dose. Scale bar-50 µm. **S2. **Resolution marks on SU-8 layers used to optimize the UV exposure dose for each layer. The required dose depends on the light source power, the features’ density, and size as well as the photoresist’s properties and thickness. a) SU-8 3005 (5µm in height), UV dose 200mJ/cm^2^ with a resolution down to 3µm (5.5 11.5µm in height). b) SU-8 3025 (11.5µm in height), UV dose 300mJ/cm^2^ with a resolution down to 20µm. Scale bar-20µm. **Fig. S3. **Electrode detachment due to poor metal-SU-8. **Fig. S4. **Strong attachment of relief gold between two electrodes due to surface tension. a) SEM image top view, Scale bar-5µm. b) Zoom-in obtained through FIB cross sectioning of the area demarcated with a white rectangle in a. Scalebar 3µm. **Fig. S5. **Resolving the Thermal stress effect on the SU-8 surface. (a) Cracks in the SU-8 surface (black arrows) resulting from thermal stress as is visualized by bright-field imaging. (b) Gradual curing and the addition of a material with nickel as an intermediate CTE value resolved the thermal stress effect. Scale bar-200µm. **Fig. S6. **Resolving the “stream-line” effects on the gold coating of the AZ photoresist in a multi-layer configuration. Images of the AZ photoresist patterned with dots sized 20-80µm and coated with Cr/Au (20/200nm). a-b) The “stream-lined” effect is clearly visible (white arrows), where an AZ line splits the circular implant, after it is concentrated in the external ring that defines the implant). c-) Good uniformity of AZ patterning is obtained by the additional steps of slow spin rate coating and full manual deposition. Scale bar - 0.5mm. **Fig. S7. **Optimization of implant release by wet etching of a sacrificial layer while preventing electrode detachment. a) A representative image of an SU-8 implant demonstrating electrode detachment following FeCl_3_ etching at room temperature for 5h. b) Electrodes are preserved intact following an optimal release process, where Nickel (Ni, 200nm) served as a sacrificial layer, Chromium (Cr, 10nm) as an adhesion layer, and HNO_3_ as an etchant at room temperature overnight. Scale bar-200nm. **Fig. S8. **XPS survey spectrum of the modified gold surface revealing the presence of RGD. In the inserts, a zoom-in on the N_1s_ and S_2p_ peaks show the presence of the amin (NH_3_) and thiol (SH) groups from the peptide on the gold surface, respectively. **Fig. S9.** SU-8 surface treatments. a-c) Contact angle measurements for various SU-8 surface treatments. a) Untreated cured SU-8 used as a control, 80^o^ C. b) N_2_ plasma-treated cured SU-8 (150W, 2min), 40^o^C) O_2_ plasma-treated cured SU-8 (150W, 2min), 7.5^o^C. d-i) PRP cell growth affected by surface treatment. d-f) 1-day post-seeding. g-i) 4 days post-seeding. a-g) Cured untreated SU-8 used as a control. c-d) N_2_ plasma (100W, 2min). e-f) O_2_ plasma (100W, 2 min). Acquired by a bright-field microscope. Scale bar-100μm. **Fig. S10. **Retinal response for current stimulation. Representative average fluorescence change highlighting successful robust stimulation induced by repetitive current stimuli administered at 0.2 Hz. The disappearance of these responses 1min after the addition of verapamil Ca^+2^ blocker to the medium (denoted by a black arrow) and their recovery following washout can be readily seen. Red stems denote the stimulation time. Scale bar-100µm. **Fig. S11. **Retinal response to high-resolution electrical stimulation. (a) Localized fluorescence changes in response to electrical stimulation of a RGC-GCaMP6f-labelled retina imaged using a 40x objective; scale bar=10µm. (b) A representative average fluorescence change (in the area denoted in red in a) induced by increasing the current amplitudes indicating an activation threshold of 0.8mC/cm^2^. (c) The overall strength-duration curve of the investigated retinal tissue. (red asterisk) and the Lapicque fit (the solid blue line). **Fig S12. **Device Biocompatibility. Cell viability, defined as the ratio between the dead cells (visualized using the proTUNNEL staining) and the overall counted nuclei (visualized using Hoechst), of ARPE cells seeded on a control surface (cover glass) and ARPE cells seeded on an implant.

## Data Availability

The data supporting the current study have not been deposited in a public repository because it was generated by various setups requiring customized analyses software in non-standard format. The data is available from the lead contact (yossi.mandel@biu.ac.il) on request.
