# Supplementary material for: Optimizing the fabrication of a 3D high-resolution implant for neural stimulation
Source: J Biol Eng. 2023 Aug 24;17:55. doi: 10.1186/s13036-023-00370-8 (PMC10463680; doi:10.1186/s13036-023-00370-8)
Supplement: Supplementary file 1 — Additional file 1: S1. UV dose effect on AZ positive photoresist patterning. a) Photoresist residues inside the AZ pattern as the result of underexposure. b) A clean AZ pattern following a sufficient exposure dose. Scale bar-50 µm. S2. Resolution marks on SU-8 layers used to optimize the UV exposure dose for each layer. The required dose depends on the light source power, the features’ density, and size as well as the photoresist’s properties and thickness. a) SU-8 3005 (5µm in height), UV dose 200mJ/cm2 with a resolution down to 3µm (5.5 11.5µm in height). b) SU-8 3025 (11.5µm in height), UV dose 300mJ/cm2 with a resolution down to 20µm. Scale bar-20µm. Fig. S3. Electrode detachment due to poor metal-SU-8. Fig. S4. Strong attachment of relief gold between two electrodes due to surface tension. a) SEM image top view, Scale bar-5µm. b) Zoom-in obtained through FIB cross sectioning of the area demarcated with a white rectangle in a. Scalebar 3µm. Fig. S5. Resolving the Thermal stress effect on the SU-8 surface. (a) Cracks in the SU-8 surface (black arrows) resulting from thermal stress as is visualized by bright-field imaging. (b) Gradual curing and the addition of a material with nickel as an intermediate CTE value resolved the thermal stress effect. Scale bar-200µm. Fig. S6. Resolving the “stream-line” effects on the gold coating of the AZ photoresist in a multi-layer configuration. Images of the AZ photoresist patterned with dots sized 20-80µm and coated with Cr/Au (20/200nm). a-b) The “stream-lined” effect is clearly visible (white arrows), where an AZ line splits the circular implant, after it is concentrated in the external ring that defines the implant). c-) Good uniformity of AZ patterning is obtained by the additional steps of slow spin rate coating and full manual deposition. Scale bar - 0.5mm. Fig. S7. Optimization of implant release by wet etching of a sacrificial layer while preventing electrode detachment. a) A representative image of an SU-8 implant dem [file 13036_2023_370_MOESM1_ESM.docx]

*Supplementary Information*

**Optimizing the Fabrication of a 3D high-resolution implant for neural stimulation**

**Gal Shpun^a,b,c^, Nairouz Farah^b,d^, Yoav Chemla^b,c,d^ , Amos Markus^b,d^ , Tamar Azrad** **Leibovitch^b,d^ , Erel Lasnoy^b,d^, Doron Gerber^d^, Zeev Zalevsky^a,c^, and Yossi Mandel^*,b,c,d,e^**

a. The Alexander Kofkin Faculty of Engineering, Bar Ilan University, Ramat Gan 5290002, Israel.

b. Faculty of Life Sciences, School of Optometry & Visual Science, Bar Ilan University, Ramat Gan 5290002 Israel.

c. Bar Ilan Institute for nanotechnology & advanced materials (BINA), Bar Ilan University, Ramat Gan 5290002, Israel.

d. The Mina and Everard Goodman Faculty of Life Sciences Bar-Ilan University, Ramat-Gan 52900, Israel

e. The Gonda Multidisciplinary Brain Research Center, Bar-Ilan University

* Author for correspondence Tel.: +972-3738-4234; [yossi.mandel@biu.ac.il](mailto:yossi.mandel@biu.ac.il)

Received: date; Accepted: date; Published: date

***UV exposure for a high aspect ratio device***

The optimization of UV exposure during a complex lithography process is affected by numerous factors and is thus challenging. Of major importance is the type of photoresist (negative or positive), the photon wavelength absorption, as well as the geometrical and mechanical characteristics of the photoresist (the thickness and aspect ratio), all of which affect the amount of energy arriving to the desired location, thus limiting the theoretical lateral resolution of the patterned features size (1,2). Furthermore, the substrate used for the fabrication or the metal electrodes, such as gold with our device, creates standing waves arising from reflections at the interface between the substrate and the photoresist, thus further limiting the lateral resolution by increasing the photoresist's wall roughness and determining the optimal exposure needed for efficient photolithography (2,3). The exposure dose ranges from underexposure to overexposure, both having different effects on the positive and negative photoresists as is shown in Fig. 3 and in Fig. S1.

In order to avoid undesired structural defects, the optimal exposure dose has to be determined for each photoresist (i.e., SU-8 3005, AZ and SU-8 3025, used for the implant base, defining the electrodes and the implant walls, respectively), while taking into account its thickness, configuration mask (e.g., design and resolution), and substrate (Ni-coated Si wafer or glass wafers, AZ onto a thin SU-8 base, and thick SU-8 on thin SU-8 over a Ni-coated Si wafer or glass wafer). To this end, manual optimization was carefully performed using the hashtag (#) symbol, as is described in Fig. S2. Using these optimization steps, we were able to achieve perfectly shaped circular micro-wells for both a positive AZ photoresist (Fig. S1b) and a negative SU-8 photoresist (Fig. 3d).

**S2. Resolution marks on SU-8 layers used to optimize the UV exposure dose for each layer.** The required dose depends on the light source power, the features’ density, and size as well as the photoresist’s properties and thickness. a) SU-8 3005 (5µm in height), UV dose 200mJ/cm^2^ with a resolution down to 3µm (5.5 11.5µm in height). b) SU-8 3025 (11.5µm in height), UV dose 300mJ/cm^2^ with a resolution down to 20µm. Scale bar-20µm.


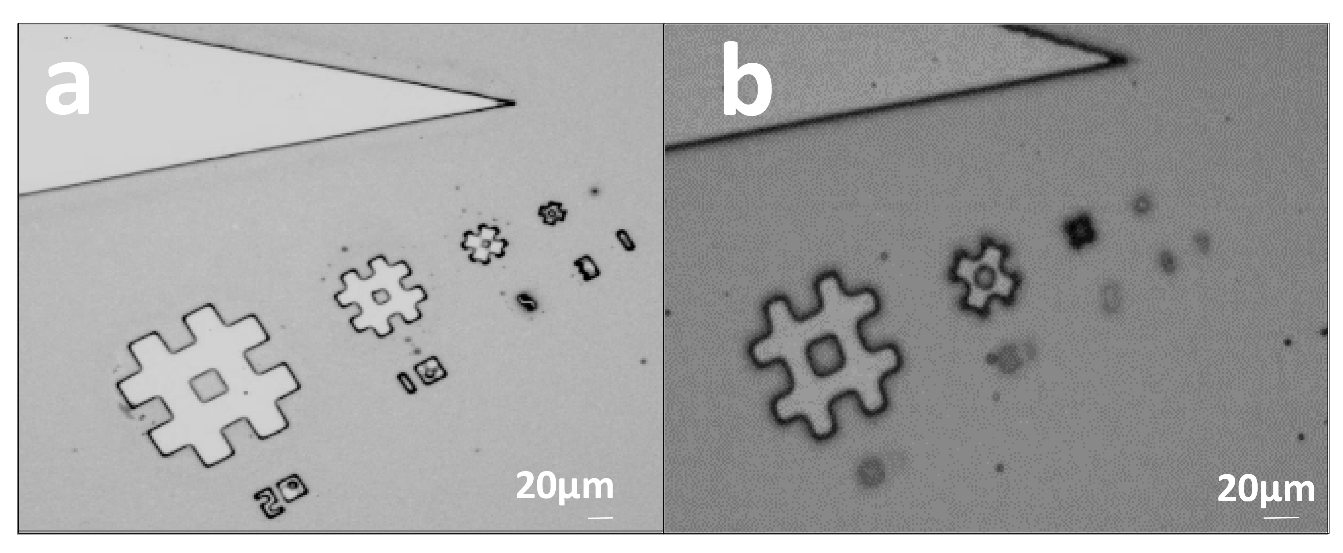


**S1. UV dose effect on AZ positive photoresist patterning.** a) Photoresist residues inside the AZ pattern as the result of underexposure. b) A clean AZ pattern following a sufficient exposure dose. Scale bar-50 µm.


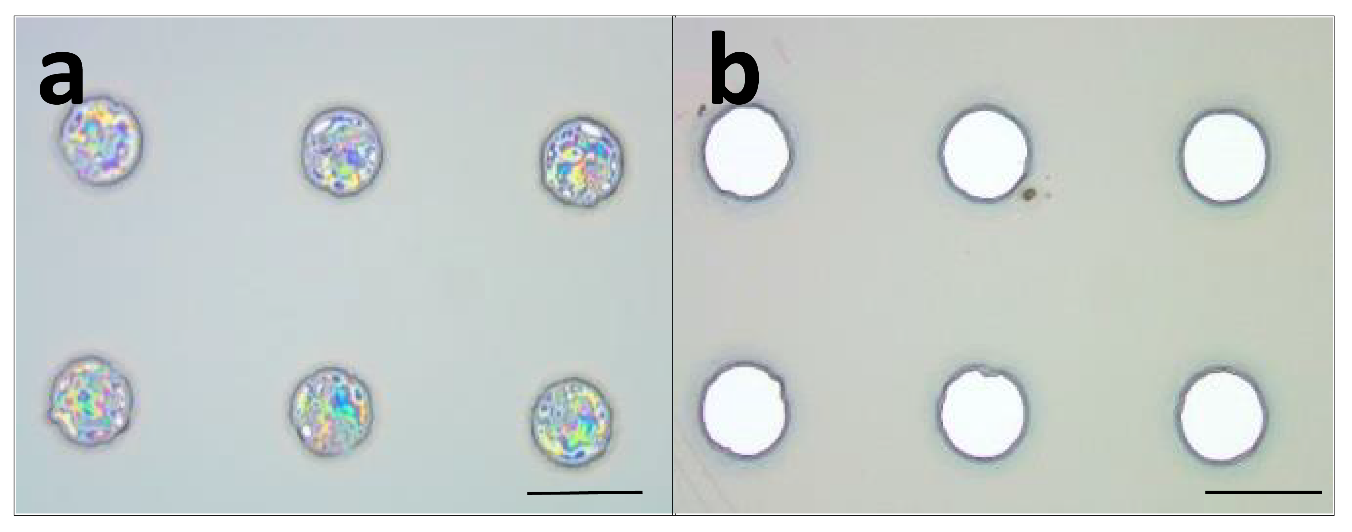


***Affecting electrode-substrate attachment***

***Poor electrode-substrate bond***

The noble properties of gold that tends not to combine with other materials together with the high hydrophobic SU-8, led to poor and weak bond between gold electrodes and SU-8 substrate, which in turn caused to electrode detachment from the implant as shown in Fig S3. This was resolved by O_2_ plasma surface treatment (150W, 3min) that increase the surface energy by breaking the SU-8 epoxy ring and the addition of a Cr or Ti adhesions layer (10nm) aims to improve the gold adhesion (see Fig. 4g in the main text).

**Fig. S3. Electrode detachment due to poor metal-SU-8 substrate.**


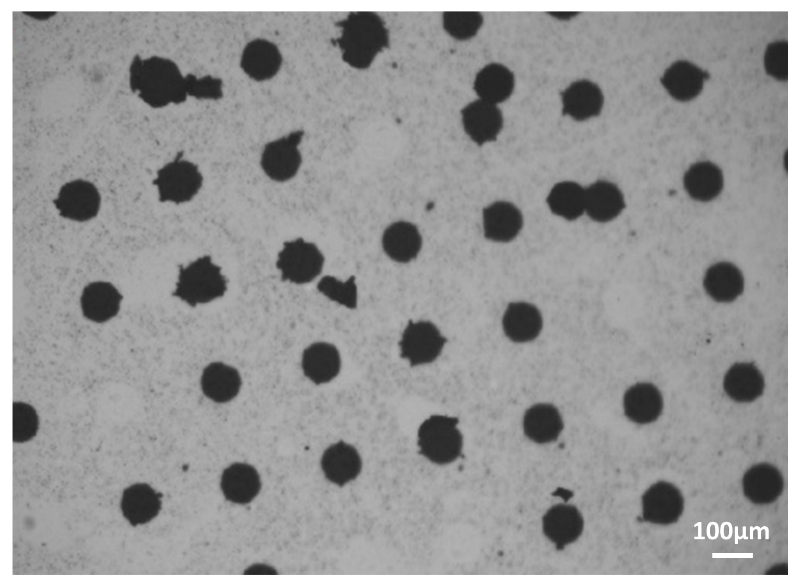


***Surface Tension***

Contrary to a negative tone photoresist, which has a “trapezii”-like structure and results in a good separation and a discontinuity in the metal layer deposited onto it, a positive tone photoresist has a “concave”-like profile, resulting in a sustained continuous deposited metal layer. Consequently, this continuity affects the electrode shape and its surface adherence due to lateral surface tensions evolving between the material’s molecules, disturbing the proceeding lift-off process and (metal detachment in defined areas), eventually leading to electrode rupturing and metal residues at its edge, as depicted in Fig. S4.

Since a conventional negative photoresist has limited resolution, we pursued a bi-layer lift-off process to resolve this issue and to obtain a good separation between the metal layer and the positive photoresist. The bi-layer process utilizes an additional layer of a fast-developing resist (e.g., PMGI, LOR) under the positive photoresist. This layer dissolves faster than the patterned photoresist during the lift-off process, therefore resulting in an "undercut" profile; thus, it efficiently separates the desired from the undesired metal regions, as in the negative "trapezii" shape.

In order to achieve the desired undercut profile, various materials with different thicknesses and dissolution rates (such as LOR10B, PMGI sf3, and PMGI sf6) were investigated. Since the dissolution rate is strongly impacted by the pre-baking temperature, the baking time, as well as the UV exposure before the photoresist coating, several application protocols were tested and optimized. In addition, we applied a second cycle of a heating step (4) to cure the patterned resist (i.e., AZ) followed by a development step to prevent its dark erosion, by reducing its sensitivity to the developer. In this second cycle, the device was heated to 120^o^C, which is higher than the AZ T_g_ but lower than that of the LOR/PMGI, such that the development contrast between these two increases, therefore enabling better control of the undercut profile while protecting the desired patterns.

**Fig. S4. Strong attachment of relief gold between two electrodes due to surface tension**. a) SEM image top view, Scale bar-5µm. b) Zoom-in obtained through FIB cross sectioning of the area demarcated with a white rectangle in a. Scalebar 3µm.


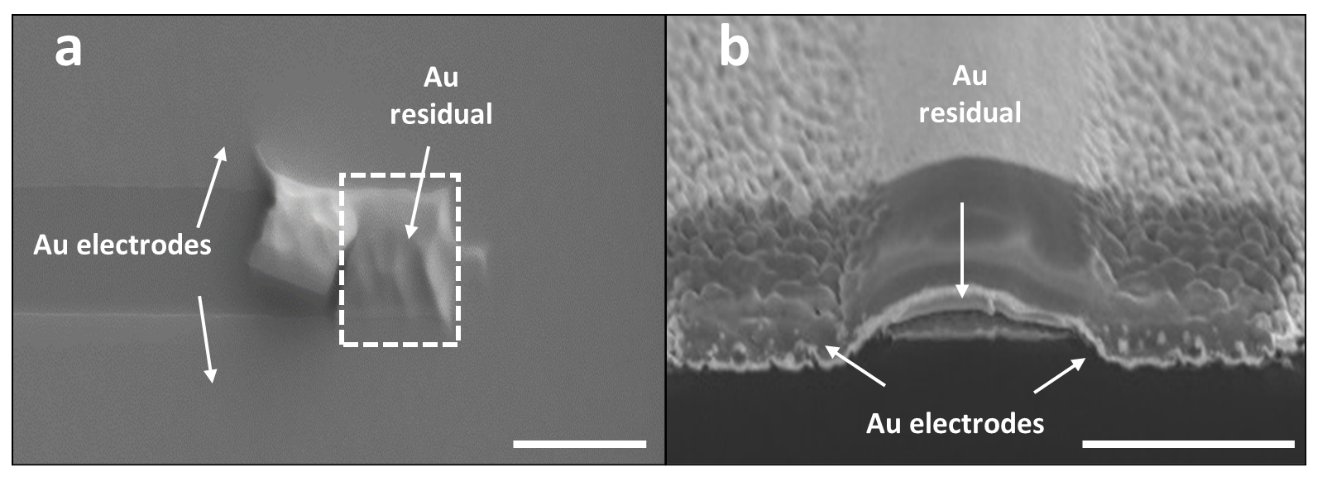


***SU-8 fabrication process: thermal stress***

Heating the material to temperatures higher than the glass transition temperature, (T_g_), is a curing step required to complete the polymerization process; it provides the polymer with its final mechanical and chemical properties and stability. The different thermal expansion coefficient (CTE) values of the SU-8 (52ppmC^-1^) and the Si wafer (2.6ppmC^-1^) may induce the development of thermal stress in the SU-8 (Fig. S5a) during this step when sharp temperature changes are applied (1,5,6). This thermal stress results in cracks in the SU-8, which is mainly observed at defect starting points such as sharp edges or areas of air bubbles that are found in polyester photomask and are transferred to the photoresist during lithography (7).

To tackle this issue, we used two strategies: First, we optimized a gradual temperature gradient from room temperature to 195^o^C at a rate of 7^o^ C min-1 and then slowly cooled down to room temperature, which is in agreement with values used by others (8–10). Second, to compensate for the mismatch between SU-8 and the silicon wafers’ CTEs, we added a Nickel-Copper layer to serve as a buffer layer with an intermediate CTE of 13.3 ppm C^-1^ (1), which reduces the thermal stress, similar to Abgrall et al. (2005) (11). This Ni layer also later serves as a sacrificial layer for releasing the device. Fig. S5b presents the optimized result of intact SU-8.

**Fig. S5. Resolving the Thermal stress effect on the SU-8 surface.** (a) Cracks in the SU-8 surface (black arrows) resulting from thermal stress as is visualized by bright-field imaging. (b) Gradual curing and the addition of a material with nickel as an intermediate CTE value resolved the thermal stress effect. Scale bar-200µm.


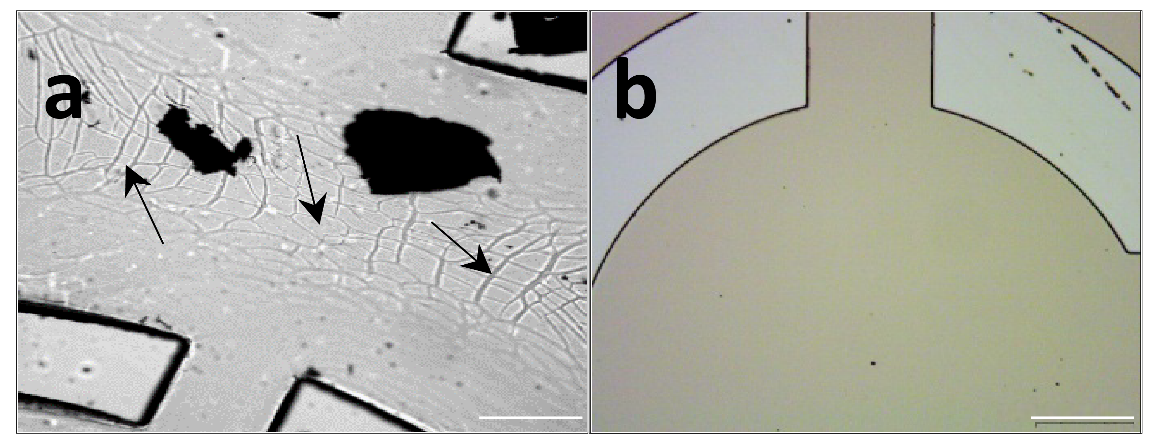


**Multilayer lithography process: streamlines and mechanical stress.**

While fabricating a 3D device via a multilayer layer-by-layer photolithography process, the cumulative effects of each of the previous steps affect the proceeding ones; this renders multilayer lithography a challenging process. One such challenge is resolving the “stream-lines” phenomenon of the second photoresist (i.e., AZ photoresist in our case, used to define the electrodes) caused by the SU-8 patterned surface (Fig. S6a-b). These "stream lines" result in an inhomogeneous surface coating by the second photoresist, which subsequently prevents the effective formation of the gold electrodes during the proceeding metallization process.

To resolve this issue, a uniform coating had to be achieved. This was accomplished by manually spreading the resist with a pipette and adding an additional spin step with low acceleration, aiming to ensure full wafer coating before speeding up to define the photoresist thickness. Implementing these steps enabled the formation of a uniformpattern at the 2^nd^ photoresist used for the electrode array formation onto the patterned SU-8, as presented in Fig. S6c.

**Fig. S6. Resolving the “stream-line” effects on the gold coating of the AZ photoresist in a multi-layer configuration.** Images of the AZ photoresist patterned with dots sized 20-80µm and coated with Cr/Au (20/200nm). a-b) The “stream-lined” effect is clearly visible (white arrows), where an AZ line splits the circular implant, after it is concentrated in the external ring that defines the implant). c-) Good uniformity of AZ patterning is obtained by the additional steps of slow spin rate coating and full manual deposition. Scale bar - 0.5mm.


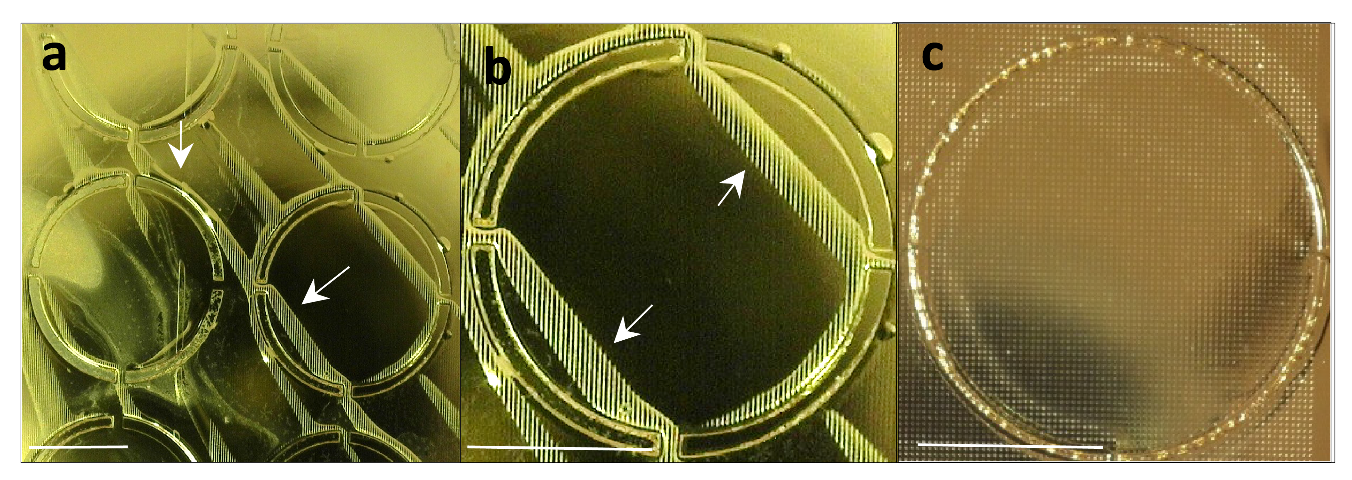


**Implant release**

Since SU-8 is an epoxy polymer, it tends to adhere to surfaces during polymerization, preventing the release of the device from most wafer substrates. In some processes, a striping technique using the commercially available Omnicoat has been introduced (11); however, it is not suitable for our complex multiple-step process. Therefore, we adopted a “sacrificial” layer approach (12), where a sacrificial metal layer is deposited onto the substrate wafer below SU-8 and is wet etched by acid at the conclusion of the fabrication process. However, since the same acid (etcher) can also etch the gold electrode or the Cr/Ti adhesion layer, as shown in Fig. S7a, the process had to be optimized to the available materials by adjusting their height contrast.

To this end, different conjugation attempts have been pursued by using Permalloy (Py), Copper (Cu), and Nickel (Ni) with both adhesion layers Ti and Cr, together at various contrast heights with diverse acid treatments (HCl, HCl+H_2_O_2_, FeCl_3_, NHO_3,_ Sigma-Aldrich, German) at various temperatures and times. We found that the best release, resulting in minimal effects on the Cr/Au electrodes (Fig. S7b), was achieved by using a 200nm Ni/Cu as a sacrificial layer wet etched by NHO_3_ 21% overnight, similar to Feiner et al. (9). The SU-8, however, gains some yellowish color, which can be prevented using ammonium peroxydisulfate salt (Merck, Germany) 10%v/v instead of the acid.

**Fig. S7. Optimization of implant release by wet etching of a sacrificial layer while preventing electrode detachment.** a) A representative image of an SU-8 implant demonstrating electrode detachment following FeCl_3_ etching at room temperature for 5h. b) Electrodes are preserved intact following an optimal release process, where Nickel (Ni, 200nm) served as a sacrificial layer, Chromium (Cr, 10nm) as an adhesion layer, and HNO_3_ as an etchant at room temperature overnight. Scale bar-200nm.


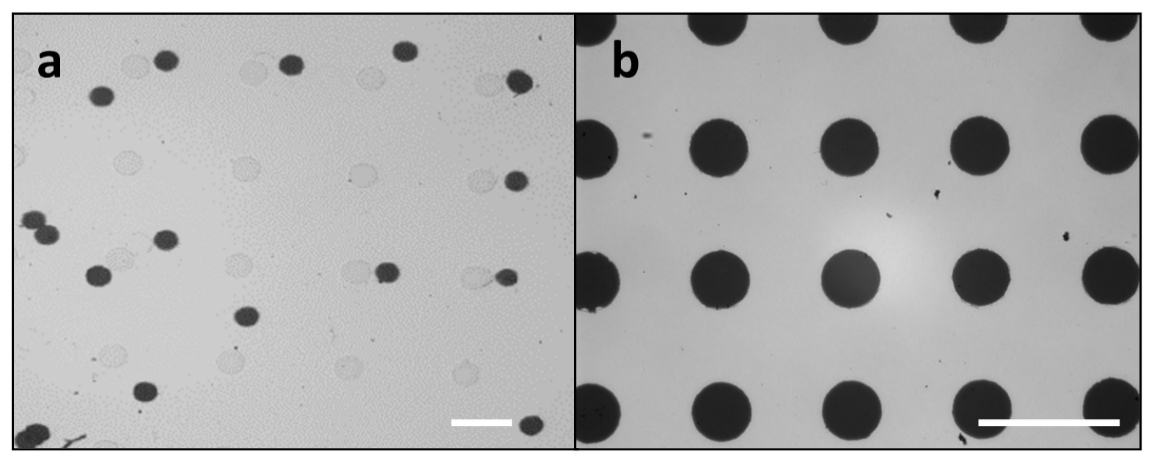


***Bio-functionalization***

1. ***X-ray photoelectron spectroscopy (XPS) analysis***

In order to evaluate the success of the biomolecule connection to the gold surface, an XPS analysis was performed (Fig. S8). Survey and high-resolution spectra were acquired at a pass energy of 80eV and 40eV, respectively. The source power was set to either 75W or 150W. The binding energies of all elements were recalibrated by setting the CC/CH component of the C1s peak at 285eV. Quantitative surface chemical analysis was performed using high-resolution core-level spectra after the removal of the nonlinear Shirley background. The measurements were carried out under UHV conditions, at a base pressure of 5x10^-10^torr (and no higher than 3x10^-9^torr). Examinations were performed on gold-coated mica glass disks (Electron Microscopy Sciences, Hatfield, PA, USA). A sulfur (S_2p_) peak from the thiol group (S atom in Cysteine) and a nitrogen (N_1s_) peak from the amine group of the peptide, indicating the presence of the RGD molecule. In addition, gold peaks, from the surface, carbon and oxygen peaks from the carboxylic acid, and peptide groups and contamination of copper from the evaporation process, were also detected.

**Fig. S8. XPS survey spectrum of the modified gold surface revealing the presence of RGD.** In the inserts, a zoom-in on the N_1s_ and S_2p_ peaks show the presence of the amin (NH_3_) and thiol (SH) groups from the peptide on the gold surface, respectively.


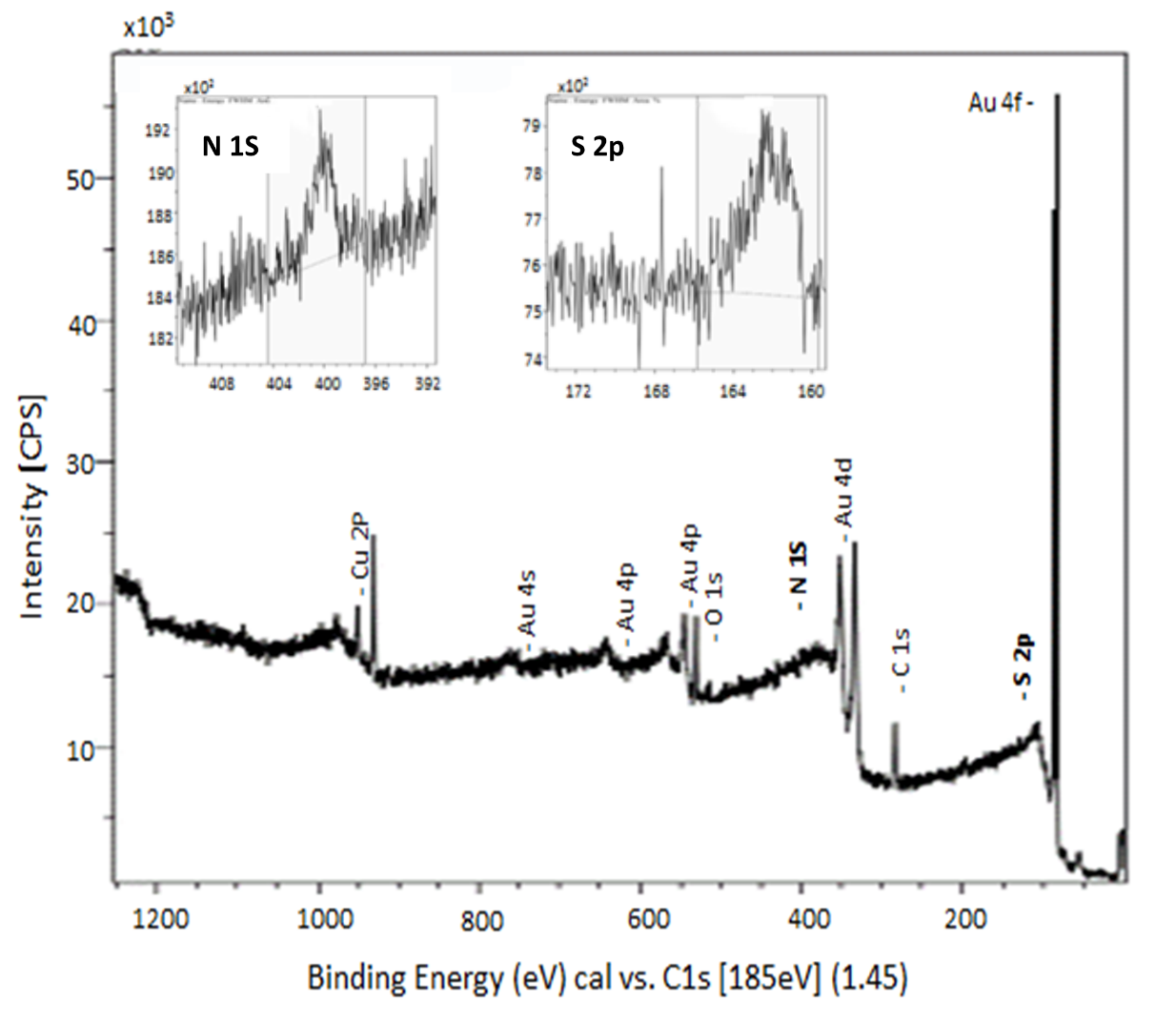


1. ***Contact angle measurement***

A drop of 5μL of DDW was placed on the center of four hard-baked SU-8 films, three of which were treated by N_2_, O_2_, and Ar dry-etching plasma (100-300W, 1-5min), whereas the fourth served as a control. The measurements were performed at 25°C and 55% moisture; Laplace-Young curve fitting was used to determine the static water contact angle values. Fig. S9 depicts representative results of contact angle measurements, which were used to assess the effect of dry-etch plasma treatment (2min, 150W) on the SU-8 surface energy and its wettability. From the measurements, it can be seen that whereas untreated SU-8 has a very high contact angle of about ~80^o^, indicating its high hydrophobicity and low surface energy, plasma-treated SU-8 has a lower contact angle (40^o^ and 7.5^o^ for N_2_ and O_2_, respectively), highlighting its superior hydrophilicity and more bio-compatible surface energy.

1. ***Effect of Surface Treatment by Plasma on Cell Adhesion***

In order to investigate the feasibility of utilizing dry-etch plasma to bio-functionalize the SU-8 surface, rPRP cells were seeded on N_2_ and O_2_-treated SU-8 films and compared to untreated film (used as a control). The confluency of the film was assessed at two time points (1- and 3-day post-seeding). Fig. S9d-i depicts the higher cellular density of N_2_ plasma-treated SU-8 over no treatment and O_2_ plasma treated surfaces.

**Fig. S9. SU-8 surface treatments.** a-c) Contact angle measurements for various SU-8 surface treatments. a) Untreated cured SU-8 used as a control, 80^o^ C. b) N_2_ plasma-treated cured SU-8 (150W, 2min), 40^o^C) O_2_ plasma-treated cured SU-8 (150W, 2min), 7.5^o^C. d-i) PRP cell growth affected by surface treatment. d-f) 1-day post-seeding. g-i) 4 days post-seeding. a-g) Cured untreated SU-8 used as a control. c-d) N_2_ plasma (100W, 2min). e-f) O_2_ plasma (100W, 2 min). Acquired by a bright-field microscope. Scale bar-100μm.


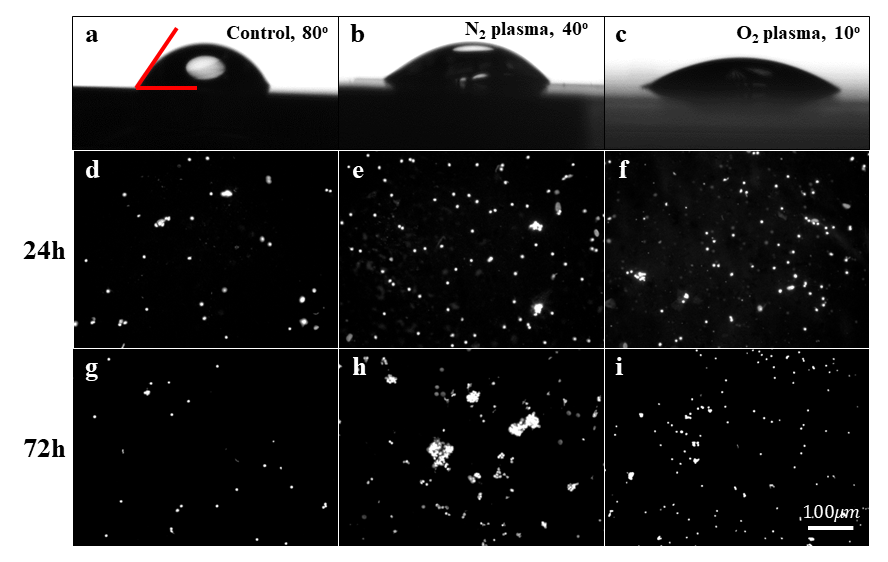


***Detailed fabrication process***

The full fabrication scheme is presented in Fig. 2 in the main text. A silicon wafer is used as the substrate (Silicon specialists, CA, USA) and is cleaned by a Piranha solution (sulfuric acid and hydrogen peroxide 3:1) for 5min, rinsed with DI water, and dried on a hot plate (Torrey, Pines scientific, Carlsbad, CA, USA) at 150^o^C in order to remove organic contamination and moisture.

1. A thin layer of nickel (200nm) is then deposited using e-beam evaporation deposition (Bestec, Berlin, Germany) to serve as a sacrificial layer.
2. A layer of SU-8, negative photoresist (5.5µm, 3005, MicroChem Corp, Newton, MA) is spin coated over the entire wafer by spin coating (3000rpm) in a humidity-controlled hood (<40%).
3. Then it is soft baked (65^o^C, 95^o^C for 3 and 7 min, respectively) and exposed to UV (210mJ/mm^2^) to form the base of the device, afterwards, it was post-exposed, developed, and cured (190^o^C, 20 min).
4. The electrodes’ formation is then performed using a bi-layer lift-off process with two steps of coating and soft-baking of LOR10B (MicroChem Corp., Newton, MA, 1μm, 5 min at 185^o^C), followed by spin coating of AZ1505 (MicroChem Corp., Newton, MA, 0.5μm, 1min at 100^o^C). The AZ photoresist is patterned by UV exposure (60mJ/mm^2^) to define the electrodes’ structure.
5. Next, a two-phase development is performed by AZ351 developer (1:4, Merck Performance Materials, Darmstadt, Germany). First, the AZ photoresist is developed for 1 min and stopped by DDW, followed by hard baking (120^o^C, 5 min) to increase the development contrast to the LOR. Next, an additional development for 1 min which stopped by DDW developed the LOR layer and created the undercut profile. Finally, the electrodes are metalized by a sequential spattering deposition of Cr/Au (10/200nm) metals, after surface cleaning by O_2_ plasma (5min, 150W, Diener, Ebhausen Germany) and argon ion-milling (10 sec).
6. Electrodes are realized by lift-off with organic solvent as NMP or DMSO at room temperature overnight.
7. An additional SU-8 (11.5µm, 3025, MicroChem Corp., Newton, MA) layer, serving as a passivation layer defining the micro-wells’ structures, is spin-coated (5000rpm) after O_2_+Ar plasma treatment (5 min, 150W) aimed at surface cleaning and layer attachment, patterned by UV exposure (350mJ/mm^2^) and cured (190^o^C, 20 min).
8. A second developing step is performed to terminate the SU-8 passivation patterning.
9. In order to release the device from the silicon substrate, the nickel layer is wet etched with nitric acid (NHO_3_, 21% w/v, sigma-Aldrich, Munich, Germany) overnight at room temperature, after which the devices are rinsed with DI three times to remove any acid residues.
10. Finally, bio-functionalization of the gold electrodes is performed by immersing the device in RGD solution (1mM, Adar Biotech, Rehovot, Israel) overnight, to stabilize the semi-covalent bonds, after which the electrodes are rinsed with DDW to remove any unbonded residues.

***Ex-vivo Retinal stimulation***

In order to investigate the feasibility of the proposed fabricated implant to stimulate retinal neurons and to serve as a subretinal implant. a model implant was fabricated on a glass substrate and used as a multi-electrode-array (MEA). Later, an isolated rat retina was mounted and electrically stimulated by the current injection system (MEA2001, Multi-Channel System Germany), and the electrically induced retinal responses were investigated (see the Methods section).

1. ***Retinal tissue preparation***

The animals were euthanized by CO_2_ inhalation and their eyes were enucleated and hemisected in Ringer’s medium (110 mM NaCl, 22 mM NaHCO_3_, 2.5mM KCl, 1.6mM MgCl, 1mM CaCl_2_, NaH_2_PO_4_, and 10mM Glucose) bubbled with a mixture of 95% O_2_ and 5% CO_2_. Using fine forceps, the vitreous humour and lens were carefully removed from the open eyecup and the retina was then hemisected and detached from the retinal epithelium.

1. ***Calcium Imaging analysis***

Calcium signals were analysed using a customized MATLAB code, similarly to our previous publication (13). Briefly, as a first step, the change in the fluorescence frame was calculated, pixel by pixel, by subtracting each acquired camera frame from the average baseline frame (calculated as the mean stimulus free frames). Next, the change in percentage for each frame was calculated by dividing the subtraction product by the average baseline frame. To evaluate the change in the cell of interest, the fluorescence changes of the relevant pixels was averaged (𝐴𝑣𝑒𝑟𝑎𝑔𝑒_𝑅𝑒𝑠𝑝𝑜𝑛𝑠𝑒). The average fluorescence change was then corrected for photobleaching (𝐶𝑜𝑟𝑟𝑒𝑐𝑡𝑒𝑑_𝐴𝑣𝑒𝑟𝑎𝑔𝑒) by either using the readily available MATLAB function *detrend*, which corrects polynomial (of any desired order) trends in the data or by fitting the data to a two-time constant exponent by the *fit* MATLAB function, followed by subtracting this fit from the data, as described in and Eq. (1):

**Fig. S10. Retinal response for current stimulation**. Representative average fluorescence change highlighting successful robust stimulation induced by repetitive current stimuli administered at 0.2 Hz. The disappearance of these responses 1min after the addition of verapamil Ca^+2^ blocker to the medium (denoted by a black arrow) and their recovery following washout can be readily seen. Red stems denote the stimulation time. Scale bar-100µm.


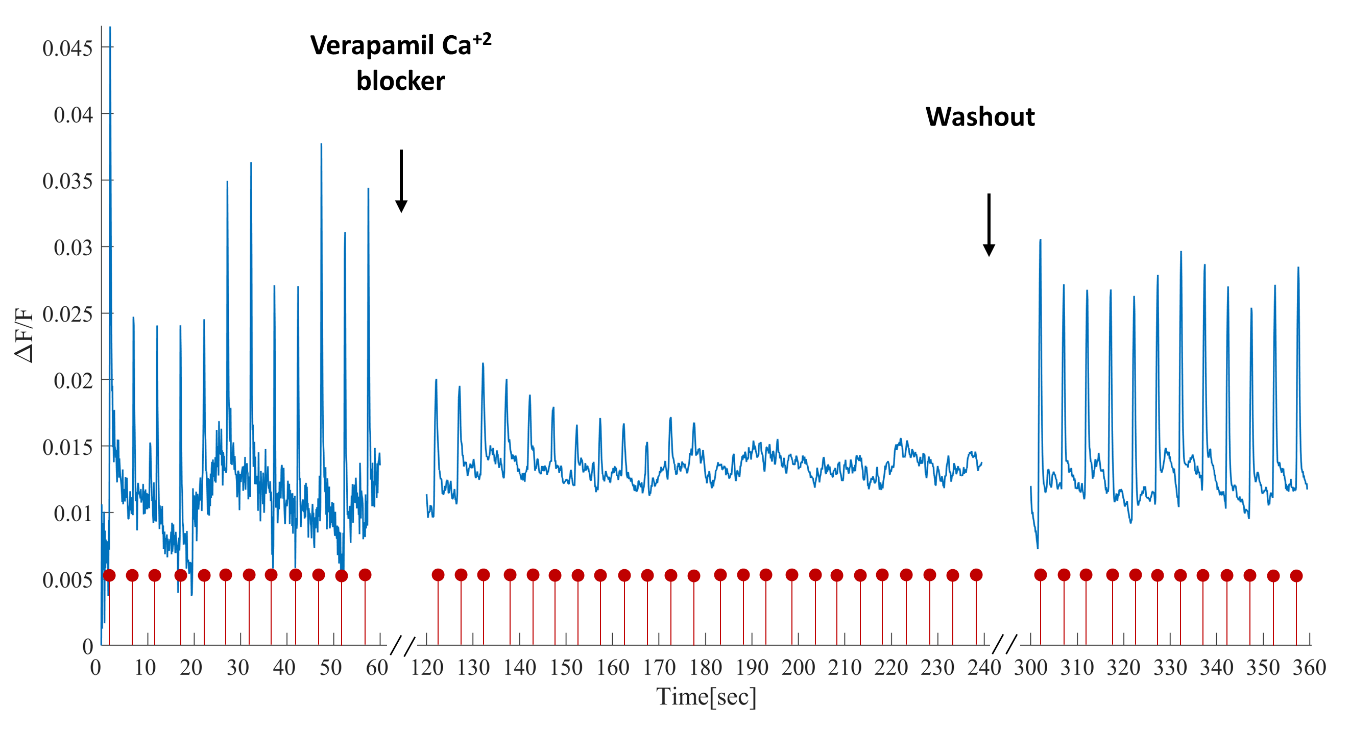


Eq. (1) $Corrected Average=Average Response-ae^{bt}-ce^{dt},$

where t is the time in seconds and a, b, c, and d are fitting parameter variables of the exponential photobleaching.

To verify that the recorded change in fluorescence was induced by calcium dynamics, a calcium blocker (Verapamil) was applied during retinal stimulation. Fig. S10 depicts that, upon application of the calcium blocker, the responses were abolished and returned, following washout, confirming that the recorded responses are induced by electrically driven calcium dynamics.

1. ***High-resolution in-vitro retinal stimulation***

Following the successful validation of in-vitro retinal stimulation using the implant protype (see the main manuscript fig. 7 and Fig. S10), we set out to investigate the feasibility of in-vitro stimulation at a higher spatial resolution. To this end, we fabricated a device with 60 electrodes on glass to enable the activation of single electrodes using the current stimulator (described above), using the same methods. A RGC GCaMP6f labelled retina was mounted on the device, as described above, and the retina was stimulated using single electrodes. Using this device, we showed the ability to stimulate the retina at a specific local area (Fig. S11a,b). Furthermore, the activation thresholds as a function of pulse duration presented the characteristic strength-duration curve (Fig. S11c).

**Fig. S11. Retinal response to high-resolution electrical stimulation**. (a) Localized fluorescence changes in response to electrical stimulation of a RGC-GCaMP6f-labelled retina imaged using a 40x objective; scale bar=10µm. (b) A representative average fluorescence change (in the area denoted in red in a) induced by increasing the current amplitudes indicating an activation threshold of 0.8mC/cm^2^. (c) The overall strength-duration curve of the investigated retinal tissue. (red asterisk) and the Lapicque fit (the solid blue line).


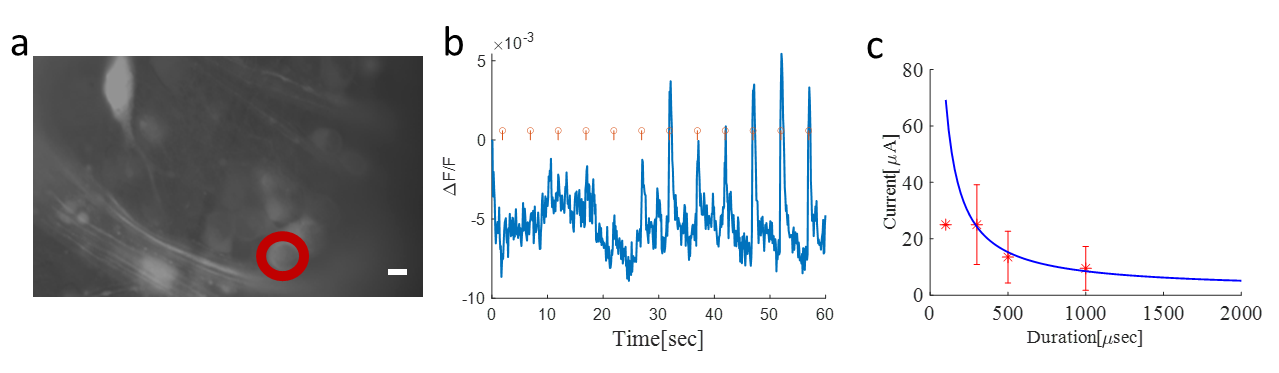


***Immuno-biological investigations Methods***

1. ***Eyecup embedding***

After enucleation, the eyes were fixated in 4% PFA for 1 hour and then rinsed with PBS. Next, the anterior segment and the lens were removed, and the eyes were embedded in increasing levels of sucrose (in PBS) from 5% to 30%. Samples were then mounted in O.C.T. Compound (Tissue-Tek® O.C.T. Compound, #4583), frozen with dry ice and kept at -80℃ at least for 24 hours. Then, 15mm cryosections were obtained at -22℃ (Leica, CM1950, [Switzerland](https://www.google.com/search?sxsrf=ALiCzsbRmVtqAA8fyevZ_aCfDkD4U_48-A:1672835233170&q=St.+Gallen&stick=H4sIAAAAAAAAAOPgE-LUz9U3MMoqM8lTgjBNTPMstbSyk63084vSE_MyqxJLMvPzUDhWGamJKYWliUUlqUXFi1i5gkv0FNwTc3JS83awMu5iZ-JgAACMo9AYWQAAAA&sa=X&ved=2ahUKEwiA3dzu9K38AhXD6qQKHd__AlEQmxMoAXoECG4QAw)). Sections were stored at -20℃ until immunofluorescent staining.

1. ***Immunofluorescent staining***

To obtain immunostaining, the slides were washed with PBS and 0.5% PBS-TWEEN (Sigma-Aldrich, St. Louis, MO) and then incubated for 1 hour in 0.01g/ml Bovine albumin (LOT M8665, MP Biomedicals™) and 5% horse serum (Biological Industries, #04-124-1A) blocker solution). The slides were then incubated overnight at 4°C with 1:1,000 goat anti-rat Iba-1 (FUJIFILM Wako Pure Chemical Corporation). Next, slides were washed with PBS and incubated for 1 hour at room temperature with 1:200 donkey anti-goat IgG (AB-ab150131, Abcam). Hoechst 1:1,000 was added and finally, 0.9% glycerol (g9012, Sigma Aldrich) with 0.1% N-propylgallate (MKCC1933, Sigma Aldrich) in PBS was used as anti-fade, followed by sealing with a cover glass. Slides were shielded from light and maintained at 4°C. Images were obtained using confocal microscopy (Leica, spectralis, Germany). The main section includes the presentation of the results in Figure 12.

***Device biocompatibility***

The biocompatibility of the fabricated device was further verified by culturing the retinal pigment epithelium on the device. The viability of the seeded cells was then visualized using the TUNEL assay and evaluated using the proTUNEL DNA fragmentation assay (GTX85584, GeneTex), which stains for apoptotic cells. To this end, 24 hours after cell seeding, the cells were fixed using 4% paraformaldehyde and stained following the manufacturer's instructions. The cells were also stained for Hoechst to visualize nuclei. Using confocal microscopy, cell viability was defined as the ratio between the number of dead cells (proTUNNEL) and the total number of all nuclei (Hoechst). Our data revealed (Fig. S12) that there was no significant difference (p>0.1) between the viability of cells seeded on the fabricated device and that of cells seeded on the control surfaces (the cover glass).

**Fig S12. Device Biocompatibility.** Cell viability, defined as the ratio between the dead cells (visualized using the proTUNNEL staining) and the overall counted nuclei (visualized using Hoechst), of ARPE cells seeded on a control surface (cover glass) and ARPE cells seeded on an implant.


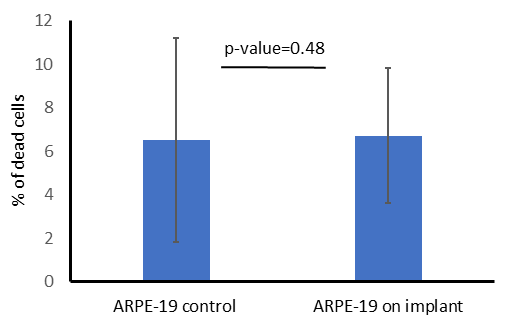


**References**

1. Rodrigo Martinez-Duarte and Marc J. Madou. SU-8 Photolithography and Its Impact on Microfluidics. In: Sushanta K. Mitra SC, editor. Microfluidics and Nanofluidics Handbook. 1st ed. Boca Raton: CRC Press; 2011. p. 231–68.

2. Christian Koch TR. Photolithography: Basics of Microstructuring. 1st ed. GmbH M, editor. Ulm: MicroChemicals GmbH; 2017. 38–149 and 174–195 p.

3. Iliescu C, Taylor H, Avram M, Miao J, Franssila S. A practical guide for the fabrication of microfluidic devices using glass and silicon. Biomicrofluidics. 2012;6(1):1–16.

4. Wilson TE, Korolev KA, Crow NA. Bilayer lift-off process for aluminum metallization. J Micro/Nanolithography, MEMS, MOEMS [Internet]. 2015;14(1):14501. Available from: http://nanolithography.spiedigitallibrary.org/article.aspx?articleid=2092198%5Cnpapers3://publication/uuid/F30C8F5B-F88A-4239-8D74-2C09693C6A55

5. P. REZAI W-IW and PRS. Microfabrication of polymers for bioMEMS. In: J.D. Cuiffi, editor. MEMS for Biomedical Applications. Draper Laboratory, USA; 2014. p. 3–45.

6. Rabih Zaouk BYP and MJM. Introduction to Microfabrication Techniques. In: Minteer SD, editor. Microfluidic Techniques - Reviews and Protocols. Totowa, NJ: Humana Press Inc.; 2006. p. 5–15.

7. Guo L, Deweerth SP. An effective lift-off method for patterning high-density gold interconnects on an elastomeric substrate. Small. 2010;

8. Feng R, Farris RJ. Influence of processing conditions on the thermal and mechanical properties of SU8 negative photoresist coatings. J Micromechanics Microengineering. 2003;

9. Feiner R, Engel L, Fleischer S, Malki M, Gal I, Shapira A, et al. Engineered hybrid cardiac patches with multifunctional electronics for online monitoring and regulation of tissue function. Nat Mater [Internet]. 2016;15(March):1–8. Available from: http://www.nature.com/doifinder/10.1038/nmat4590

10. Kilchenmann SC, Rollo E, Maoddi P, Guiducci C. Metal-Coated SU-8 Structures for High-Density 3-D Microelectrode Arrays. 2016;25(3):425–31.

11. Abgrall P, Lattes C, Conédéra V, Dollat X, Colin S, Gué AM. A novel fabrication method of flexible and monolithic 3D microfluidic structures using lamination of SU-8 films. J Micromechanics Microengineering. 2005;16(1):113–21.

12. Tatikonda A, Jokinen VP, Evard H, Franssila S. Sacrificial layer technique for releasing metallized multilayer SU-8 devices. Micromachines. 2018;

13. Schick R, Farah N, Markus A, Korngreen A, Mandel Y. Electrophysiologic characterization of developing human embryonic stem cell-derived photoreceptor precursors. Investig Ophthalmol Vis Sci. 2020 Sep 1;61(11).
